# Monolithically integrated silicon photonic biaxial FMCW LiDAR system

**DOI:** 10.1038/s41467-026-77490-4

**Published:** 2026-09-04

**Authors:** Mathias Prost, Huaqing Qiu, Guillaume Croes, Manuel Reza, Jac Romme, Rainer Hornung, Javier Pérez Santacruz, Elbert Bechthum, Ziduo Lin, Jochem Govers, Pawel Bembnowicz, Brecht Berteloot, Esteban Venialgo, Erik Emmen, Felipe Pacheco de Oliveira, Shahzad Muzaffar, Evgenii Tiurin, Paul Mateman, Nicolas Chauvet, Peter Girouard, Tangla D. Kongnyuy, Mennatallah Kandil, Maliheh Ramezani, Puvendren Subramaniam, Padraic E. Morrissey, Sean Collins, Matthew L. Hall, Peter O’Brien, Noor Schilder, Christian Bachmann, Joost Brouckaert, Ruud Oldenbeuving, Dongjae Shin, Roelof Jansen, Peter Gerets, Marcus Dahlem

**Affiliations:** 1 imec, Kapeldreef 75, 3001 Leuven, Belgium; 2 imec Netherlands, HTC31 5656 AE, Eindhoven, Netherlands; 3 Tyndall National Institute, Cork, T12 R5CP Ireland

**Keywords:** Imaging and sensing, Silicon photonics, Integrated optics

## Abstract

Three-dimensional sensing is critical for autonomous systems, robotics and industrial monitoring, motivating compact frequency-modulated continuous-wave (FMCW) LiDARs, which optically measure distance and velocity. However, current integrated designs using optical phased arrays (OPAs) for beam steering or focal-plane arrays (FPAs) for parallel reception face trade-offs in optical power, aperture and acquisition speed. Here we show a biaxial FMCW LiDAR that monolithically integrates an OPA transmitter and coherent FPA receiver with over 4500 functional components on separate optical paths, eliminating the need for circulators. A line-shaped beam enables agile steering, while a lens-assisted FPA provides a large receiving aperture and parallel single-wavelength readout. The system images at 20 m using 3.7 mW of emitted power, with 5 cm depth resolution and 1.4 mm/s velocity resolution. It produces point clouds at 10 frames per second (fps) and supports acquisition rates up to 41 fps, offering a compact and practical route towards integrated FMCW LiDAR.

## Abstract (web summary)

The authors demonstrate a compact solid-state LiDAR chip that combines beam steering and parallel detection, enabling three-dimensional imaging at 20 m with high depth and velocity resolution, and up to 10 frames per second.

## Introduction

Advanced sensing techniques enable interaction between autonomous systems and three-dimensional (3D) environments^1–4^. Among various sensing modalities, frequency-modulated continuous-wave (FMCW) LiDAR provides accurate geometric and depth information and operates robustly under both strong ambient illumination and complete darkness, enabling widespread deployment in robotics, aerial platforms, and autonomous vehicles. Compared to mechanical LiDAR systems relying on rotating or galvanometric scanners^5,6^, solid-state LiDAR eliminates moving parts and offers improved stability, extended operational lifetime, and maintenance-free operation^3,4^. FMCW LiDAR exploits coherent detection to simultaneously extract range and velocity information with high sensitivity, providing enhanced robustness against background noise compared to time-of-flight approaches^7^.

Recent advances in photonic integrated circuits (PICs) have enabled increasingly integrated and high-performance FMCW LiDAR systems. In such systems, solid-state beam steering and spatial sampling are predominantly realized using either optical phased arrays (OPAs)^8–17^ or focal-plane arrays (FPAs)^18,19^. OPAs are particularly attractive as transmitters, as they enable power scaling through coherent far-field beam combining^20^ and provide agile, programmable beam steering with high angular resolution^9,11,13,14^. However, extending OPAs to two-dimensional (2D) beam steering remains challenging. Existing approaches either combine a one-dimensional (1D) OPA with wavelength tuning^11,21^ or employ fully 2D-OPAs^22,23^. Within the framework of FMCW LiDAR, the former requires wavelength sweeps spanning hundreds of nanometers to achieve a large field of view (FOV), which is highly challenging for coherent FMCW operation, while the latter typically incurs substantial insertion loss, leading to a degraded signal-to-noise ratio (SNR). In addition, the point-by-point scanning paradigm intrinsic to most OPA-based architectures fundamentally limits the achievable frame rate^13,16^. Beyond static OPAs, alternative approaches exploiting acousto-optic modulation have demonstrated an elegant route toward dynamically reconfigurable grating emitters, enabling single-wavelength 2D beam steering^12,24^. At present, however, these schemes generally rely on relatively high radio-frequency drive power, posing challenges for scalable and energy-efficient integration.

By contrast, FPA-based LiDAR systems employ a lens-assisted optical interface that maps each pixel of a photonic receiver array to a distinct direction in the far-field^18,19^. This approach enables 3D imaging at a single wavelength, avoiding the laser complexity associated with wide wavelength tuning for FMCW LiDAR applications. The effective receiving aperture of FPAs assisted by a lens is typically an order of magnitude larger than that of OPAs, providing clear advantages for long-range detection. In contrast, the relatively small aperture of typical OPAs makes them less favorable as receivers for long-range coherent sensing. However, in coherent FMCW implementations, 2D spatial addressing in FPA-based systems is inherently tied to photonic switching and pixel-level electronic readout, such that increasing spatial resolution requires a proportional increase in coherent receiver channels and signal readout and processing complexity.

Here we demonstrate a solid-state biaxial FMCW LiDAR architecture that monolithically integrates an OPA transmitter with a coherent FPA receiver on a silicon photonics platform, comprising over 4500 photonic functional components integrated on a single chip. The spatial separation of transmission and reception eliminates the need for an optical circulator, alleviating a key constraint for scalable photonic integration^18,25^. More fundamentally, this architecture brings together the complementary strengths of the two subsystems, uniting the high power-handling capability and programmable beam steering of the OPA with the large effective aperture and parallel coherent acquisition of the FPA. Moreover, the precise angular control afforded by the OPA increases transmitter-side flexibility, facilitating more robust spatial alignment between the reflected signal and the FPA receiver array, and offering a new perspective for the design of solid-state LiDAR systems.

Experimentally, the engineered line-shaped far-field of the OPA supports line-by-line beam scanning that is collected by the FPA receiver, enabling parallel point cloud acquisition across the array. Using this approach, we demonstrate single-wavelength 3D imaging of a target at distances up to 20 m with an emitted optical power of 3.7 mW. The measured depth and velocity accuracies reach 5 cm and 1.4 mm/s, respectively. Benefiting from the line-by-line scanning scheme, the prototype experimentally achieves parallel point cloud acquisition at 10 frames per second (fps), currently limited by data transfer and computer-based processing throughput. Based on the timing budget, an optimized embedded implementation that eliminates the computer-side point cloud reconstruction and 3D visualization overhead can support frame rates of up to 41 fps.

## Results

### Scalable biaxial LiDAR architecture

**Fig. 1:**
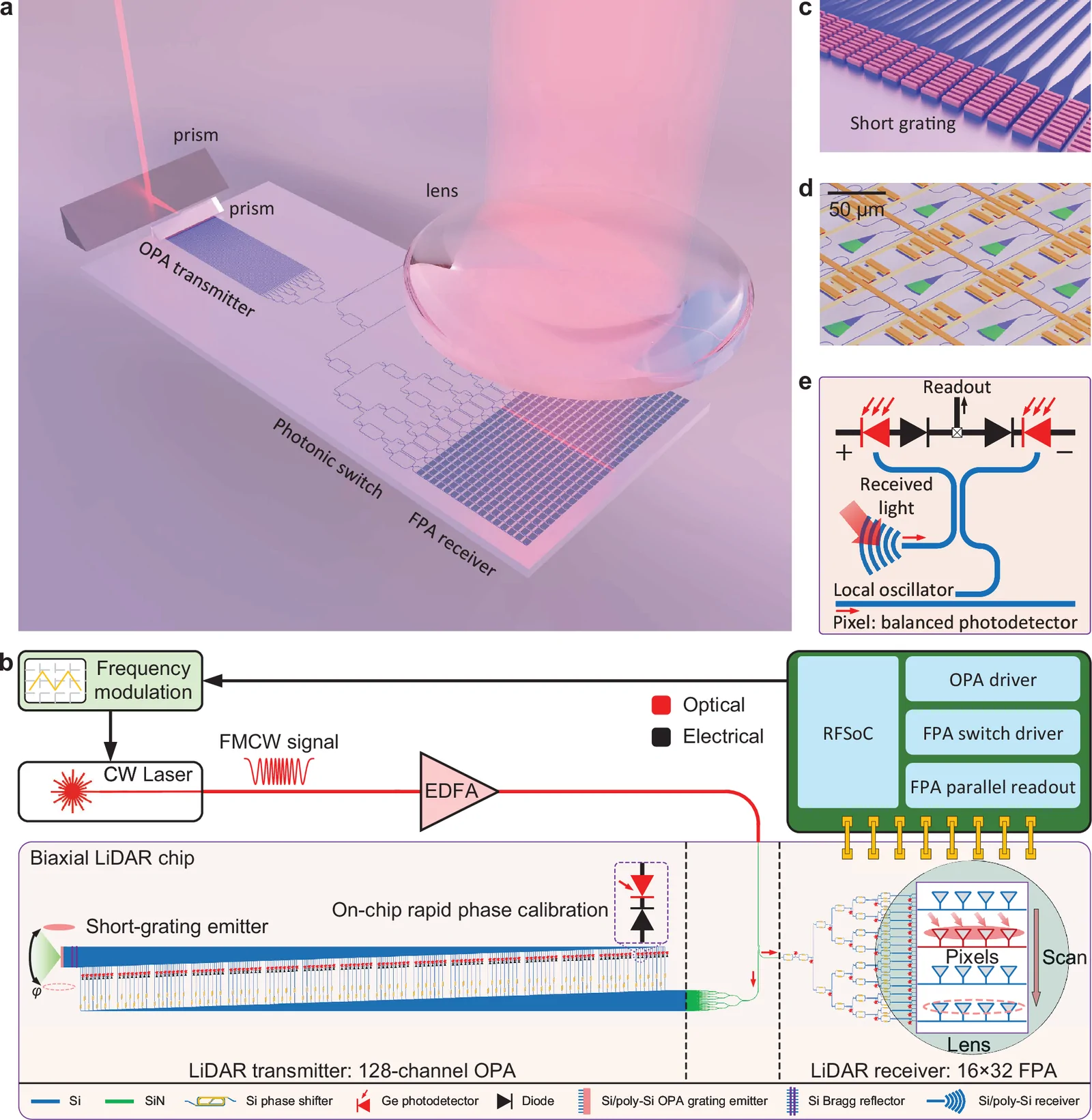
Solid-state biaxial LiDAR architecture. **a** Schematic of the monolithically integrated LiDAR chip. **b** Experimental setup and detailed PIC structures comprising an OPA transmitter and an FPA receiver. The photonic chip is designed with multiple waveguide materials to optimize the overall system performance. The 128-channel OPA transmitter enables low electrical power consumption, high optical power handling, rapid on-chip phase calibration, and a high main-lobe to total emitted power ratio. The FPA receivers contain a 32 × 16 heterodyne detector array. **c** Zoomed-in view of the short grating emitters in the OPA, generating a line-shaped far-field pattern for rapid line-by-line scanning. **d** Zoomed-in view of the heterodyne detector array, with one BPD per pixel. **e** Schematic of the heterodyne detector pixel. OPA optical phased array, FPA focal plane array, LiDAR light detection and ranging, CW continuous-wave, FMCW frequency-modulated continuous-wave, EDFA erbium-doped fiber amplifier, RFSoC radio-frequency system-on-chip, Si silicon, SiN silicon nitride, Ge germanium. **b**, **e** are reprinted with permission from IEEE Proceedings.

Figure 1a presents the schematic of the monolithically integrated biaxial LiDAR, comprising a silicon photonic chip, an imaging lens, and two prisms for fixed angular offset. As shown in Fig. 1b, the chip incorporates an OPA-based transmitter and an FPA-based coherent receiver as separate modules, forming a biaxial architecture^26^. The OPA steers the transmitted beam toward the far-field target. The reflected light is then collected by the imaging lens and mapped onto the receiver pixels in the FPA, which are addressed row by row through photonic and electrical switches. By combining 1D-OPA beam steering with 2D-FPA reception at a fixed wavelength, the system enables full 3D imaging without wavelength tuning, removing the need for broadly tunable FMCW lasers spanning hundreds of nanometers, while preserving the fast beam-steering capability of phase-based modulation for line-by-line scanning. The experimental setup, also shown in Fig. 1b, uses a continuous-wave laser that is frequency modulated to generate the FMCW waveform and coupled into the photonic chip, which integrates a 128-channel OPA transmitter and a 32 × 16 coherent FPA receiver. By design, 90% of the laser power is directed to the OPA for transmission, while the remaining 10% is delivered to the FPA to serve as the local oscillator (LO), enabling shot-noise-limited coherent detection^27^.

As shown in Supplementary Fig. 1a–d, each OPA channel has a low-power thermo-optic phase shifter, providing beam steering with an efficiency of 6.4 mW/π phase shift and a modulation bandwidth of 12.5 kHz^13,28^. Integrated phase interrogators placed between adjacent channels enable rapid on-chip phase calibration. In the current implementation, the calibration requires approximately 180 ms per channel to sweep 32 voltage points and extract the phase-voltage relationship, with the present limitation mainly imposed by the data readout capability^29,30^. In principle, the calibration time can be further reduced to roughly 10 ms. It is important to note that the calibration algorithm does not run continuously during normal data acquisition. Beam steering relies on a pre-calibrated look-up table, and re-calibration is only performed during system initialization or if performance degradation is observed. Therefore, the above calibration times do not affect the normal operation of the LiDAR system.

To reduce the routing complexity, the 128 OPA channels are divided into 8 blocks of 16 channels and addressed using a cross-point scheme, reducing the number of bus metal wires from 256 (128 × 2) for independent phase-interrogator control to 24 (16 + 8). In addition, integrated Si diodes provide electrical isolation by suppressing sneak-current paths and dark-current leakage from unselected photodiodes, thereby mitigating inter-channel crosstalk, as illustrated in Supplementary Fig. 1e. Therefore, By tuning the phase shifters, the OPA far-field pattern is calibrated into a bright beam, as shown in Supplementary Fig. 2a.

The 128-channel OPA transmitter has a 2.94 μm pitch, providing a FOV of 30°. Although each grating antenna is 50 μm long, most of the optical power is diffracted into free space within the first 10 μm, resulting in an effective near-field aperture of 376 μm  × 10 μm. Under the Fraunhofer approximation, the far-field response follows:

1 $$E(\theta,\varphi )=AF(\theta,\varphi )\cdot EF(\theta,\varphi )$$

where *AF* and *EF* represent the array and element factors, respectively, and *θ* and *φ* denote the elevation and azimuth angles^13^. The simulated OPA far-field pattern is shown in Supplementary Fig. 2b, with a 3 dB beamwidth of 0.23° × 7.9°, as shown in Supplementary Fig. 2c, d, respectively. In addition, widening the grating antenna to 2.7 μm suppresses the grating lobes and increases the fraction of optical power in the main lobe, thereby reducing the effective OPA loss. As shown in Supplementary Fig. 2c, approximately 74% of the emitted optical power is confined to the main lobe within the 16° working FOV of the LiDAR prototype, which is defined by the FPA size and the imaging lens.

After being launched into free space by the OPA transmitter and reflected from the target, the FMCW signal is collected by an imaging lens and focused onto a selected row of the FPA receiver, as shown in Fig. 1b. The FPA incorporates a reconfigurable 1 × 32 photonic switch implemented as a Mach-Zehnder interferometer (MZI) tree and a pixel array with a pitch of 75 μm (Fig. 1d). The LO signal is sequentially routed to activate the receiver rows as the line-by-line scanning proceeds. Each pixel (Fig. 1e) comprises a grating antenna and a balanced photodetector (BPD). The BPD consists of a 50/50 coupler that mixes the received signal with the LO, two germanium-silicon (Ge/Si) photodiodes (PDs), and two silicon diodes for cross-point addressing, enabling heterodyne detection for extracting distance and velocity information^26^. As shown in Supplementary Fig. 3a–c, the receiving inclination angles are varied across channels to match the chief-ray angular range of the lens, but are kept identical across rows within each channel to preserve the polarization alignment with the line-shaped OPA emission. This design reduces polarization mismatch with the transmitter while enabling each channel to collect light over the appropriate angular range, thereby increasing the effective aperture. The grating pitches were designed for a receiving angular range of  − 4° to 4°, but fabrication-induced deviations shift the actual range to approximately  − 5° to 5° with only minor additional optical loss. Although this configuration results in increased back-reflection, most prominently in the central channels with grating emission angles of  ± 1.5°, this does not affect their operation as receivers for free-space coupling into the photonic chip.

As illustrated in Supplementary Fig. 4, heterodyne detection is enabled by aligning the received optical signal and the LO to the same row in the receiver. The corresponding metal interconnect for this row is enabled, while all other rows remain inactive to suppress inter-row crosstalk. The 16 BPDs within the active row are reverse-biased and operated in parallel, and the resulting differential photocurrents are read out simultaneously. Integrated silicon diodes within each BPD provide electrical isolation and enable cross-point addressing, thereby suppressing inter-channel crosstalk while supporting efficient signal multiplexing. Consequently, the number of required electrical I/O connections for the FPA receiver is reduced from an *N*^2^ to an *N*-level scaling, substantially enhancing system scalability.

### Opto-electronic packaging for prototype

**Fig. 2:**
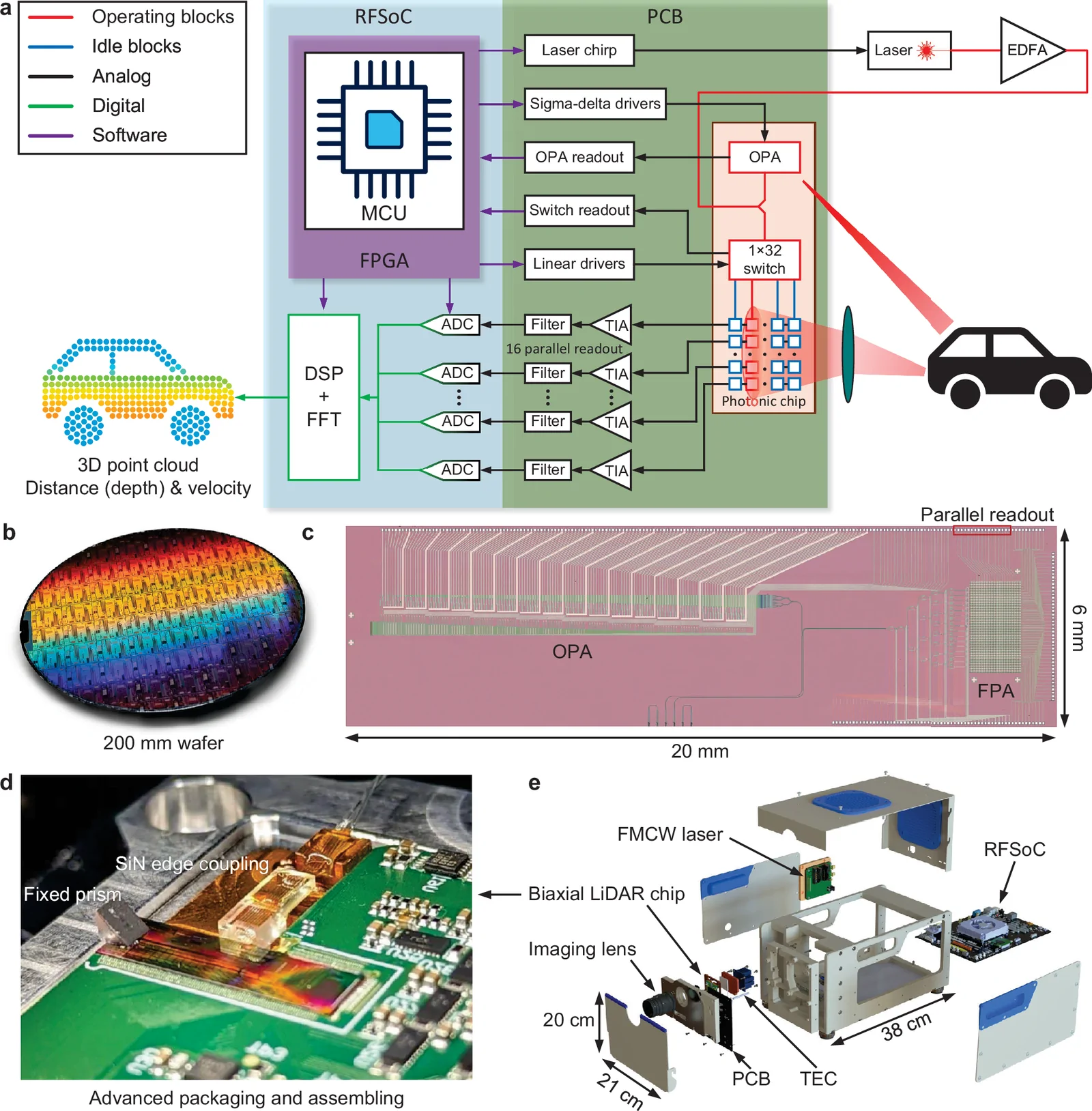
Wafer-scale LiDAR photonic chip and prototype packaging. **a** System-level illustration of the LiDAR prototype. **b** Image of the fabricated 200-mm LiDAR photonic wafer, containing more than 135 functional LiDAR photonic chips. **c** Image of the biaxial LiDAR chip with a footprint of 20 mm × 6 mm. **d** Packaged photonic chip incorporating a fiber array coupled to SiN edge couplers and two fixed prisms for static beam-direction adjustment. **e** Exploded view of the fully biaxial LiDAR prototype system. RFSoC radio-frequency system-on-chip, MCU microcontroller unit, FPGA field-programmable gate array, PCB printed circuit board, OPA optical phased array, FPA focal plane array, FMCW frequency-modulated continuous-wave, EDFA erbium-doped fiber amplifier, TIA transimpedance amplifier, ADC analog-to-digital converter, DSP digital signal processing, FFT fast Fourier transform, TEC thermoelectric cooler.

The system schematic of the LiDAR prototype is shown in Fig. 2a. A radio-frequency system-on-chip (RFSoC) is employed for global control, signal synchronization, and digital signal processing (DSP). The OPA phase shifters are driven using sigma-delta modulation, which reduces the electrical drivers and lowers overall system power consumption. All 16 pixels in the active row simultaneously deliver electric signals to the discrete analog front-end (AFE). The photocurrents are first converted to voltages by transimpedance amplifiers (TIAs). The resulting signals are then anti-aliasing filtered by a third-order inverse Chebyshev low-pass filter with a cutoff frequency of 30 MHz, digitized by analog-to-digital converters (ADCs) in the RFSoC, and subsequently processed using fast Fourier transform (FFT)-based DSP to extract distance and velocity information. Benefiting from parallel acquisition, the line-by-line scanning enabled by spatial multiplexing achieves a 16-fold increase in effective scanning speed compared to conventional point-by-point OPA scanning.

The biaxial LiDAR PICs are fabricated on a commercial 200-mm CMOS-compatible silicon photonics platform, with a total chip area of 20 mm × 6 mm, as shown in Fig. 2b, c. The zoomed-in microscope images of the LiDAR components are shown in Supplementary Fig. 5. The chip is subsequently optically packaged with a fiber array and a prism on a printed circuit board (PCB) at Tyndall National Institute^31^, together with the necessary electronic components, as illustrated in Fig. 2d. The complete module, including the PICs, FMCW laser source, imaging optics, RFSoC, and thermal control unit, is then assembled using advanced opto-electronic packaging to form a compact, fully integrated LiDAR system prototype.

### Rapid 3D imaging and velocimetry

The on-chip OPA transmitter and FPA receiver exhibit insertion losses of approximately 12 dB and 8 dB, respectively, mainly arising from waveguide routing loss and grating-related loss. These losses are expected to be significantly reduced with further process and design optimization. With 60 mW of optical power injected into the silicon nitride (SiN) bus waveguide, the OPA delivers approximately 3.7 mW into free space as a line-shaped beam. The FMCW chirp is generated by direct modulation of a commercial continuous-wave laser and linearized through an iterative calibration procedure, as shown in Supplementary Fig. 6.

**Fig. 3:**
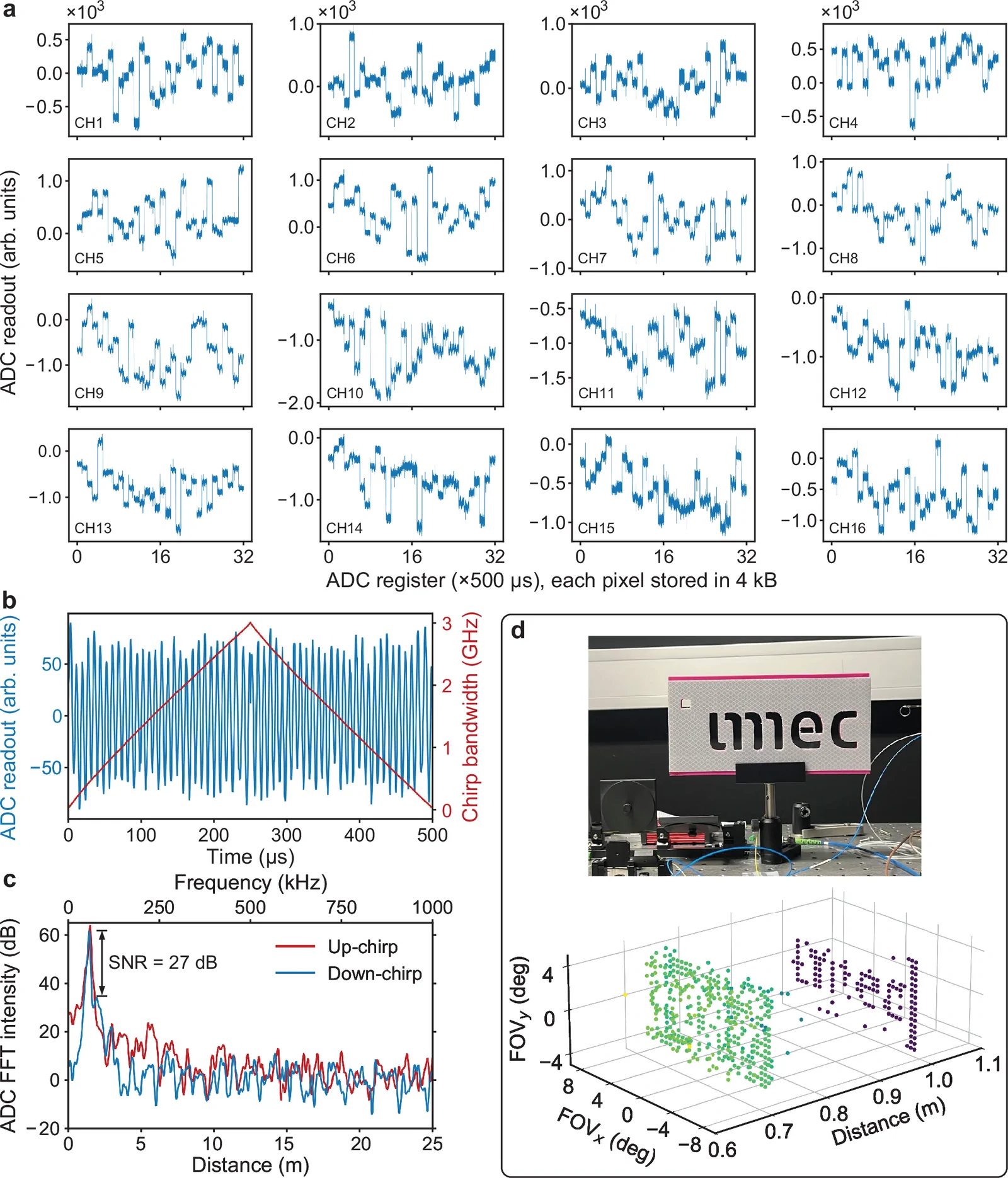
Characterization of 3D imaging results. **a** Full-frame readout data. 16 channels are read out simultaneously during line-by-line scanning across 32 rows, with the readout data of each channel stored in a 4 kB register. **b** Pre-distorted triangular chirp waveform with a duration of 500 μs and a maximum bandwidth of 3 GHz, and a representative pixel read-out signal. Each 500 μs triangular chirp waveform is sampled with 2048 points. **c** Representative FFT spectrum from a receiver pixel, indicating a target distance of 1 m and an SNR of 27 dB. The up-chirp and down-chirp peaks overlap due to the static target. **d** Reconstructed 3D imaging of the “imec” logo at 1 m. ADC analog-to-digital converter, FFT fast Fourier transform, FOV field of view. **a** and **d** are reprinted with permission from IEEE Proceedings.

To demonstrate 3D imaging, an “imec” logo target is placed at a distance of 1 m. The acquired full-frame data are shown in Fig. 3a, comprising simultaneous outputs from 16 channels across 32 rows^32^. For each pixel, the temporal waveform is recorded in a 4 kB ADC register using a 500 μs triangular chirp waveform, sampled with 2048 points (Fig. 3b). With a maximum chirp bandwidth of 3 GHz, the resulting depth resolution is approximately 5 cm. Figure 3c shows the measured distance profile for a receiver pixel from a static target located at 1 m. The reflective target is made of a material similar to that used in traffic signs^18^. Using a compact imaging lens with an 8-mm focal length (*f*/1.4), the system achieves a FOV of 16° × 8°. The line-shaped OPA pattern is imaged onto the pixel array with a calculated footprint of 35 μm × 1.2 mm, set by the image lens, the effective near-field aperture of 376 μm × 10 μm and the 0.23° × 7.9° beamwidth. The 7.9° beamwidth matches the 8° FOV, so the 1.2-mm length precisely spans the selected row, while the 35-μm width fully covers the grating receivers, resulting in a fill-factor loss of approximately 12 dB. The reconstructed 3D image is shown in Fig. 3d. Enabled by line-by-line scanning through spatial multiplexing, the LiDAR prototype experimentally demonstrates a frame rate of 10 fps, which is currently limited by the data-transfer and computer-based processing throughput. However, the architecture theoretically supports acquisition rates of up to 41 fps, even when using relatively slow thermo-optic phase shifters^13,16,28^.

**Fig. 4:**
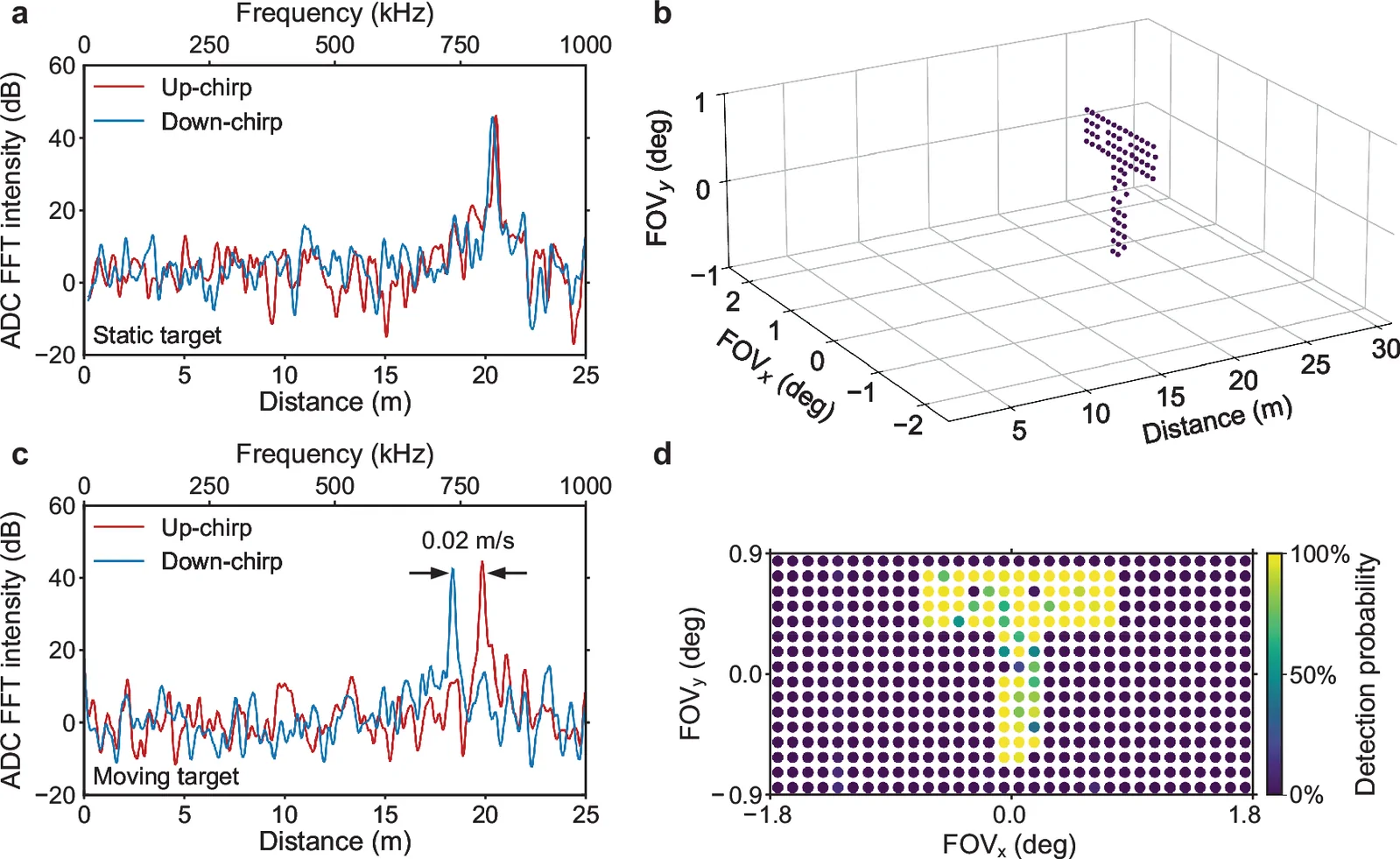
Characterization of 20 m ranging and Doppler velocimetry. **a** FFT spectrum acquired from a representative receiver pixel, revealing the beat-frequency peak corresponding to a static “T” target positioned at a distance of 20 m. **b** Reconstructed point cloud of the “T” target from a single, non-averaged frame. **c** FFT spectrum from a representative receiver pixel for a moving target with a velocity of 0.02 m/s. The target is moving away from the LiDAR prototype, from 19 m to 20 m, as indicated by a higher beat frequency in the up-chirp compared with the down-chirp. **d** Detection probability evaluated over 100 accumulated frames, demonstrating robust target detection. ADC analog-to-digital converter, FFT fast Fourier transform, FOV field of view.

The long-range performance of the system is next evaluated using a “T” logo target positioned at 20 m. Measurements are performed with a 35 mm focal-length lens (f/1.4) and a triangular chirp waveform of 1000 μs which is sampled with 4096 points, yielding a FOV of 3.6° × 1.8°. The distance profile of the static target after FFT processing is shown in Fig. 4a, and the reconstructed three-dimensional image at 20 m from a single non-averaged frame is shown in Fig. 4b, displaying only detected points, with the FFT peak 25 dB above the baseline.

The velocity is characterized by moving the target towards the LiDAR prototype. The Doppler-induced frequency separation between the up-chirp and down-chirp enables the extraction of a velocity of 0.02 m/s using

2 $$v=\frac{\lambda }{4}\left({f}_{{{{{\rm{up}}}}}}-{f}_{{{{{\rm{down}}}}}}\right)$$

where *f*_up_ and *f*_down_ denote the up- and down-chirp beat frequencies, with *f*_up_ > *f*_down_ indicating a moving away target (Fig. 4c)^19^. In order to evaluate the detection probability, 100 frames were accumulated, as shown in Fig. 4d, demonstrating a high detection probability at 20 m.

The velocity resolution was further evaluated using a rectangular board mounted on a rotating stage to generate spatially varying motion. As shown in Supplementary Fig. 7, velocity differences of approximately 1.36 ± 0.08 mm/s were clearly resolved. This performance is consistent with the theoretical velocity resolution of approximately 0.8 mm/s, given by the FMCW Doppler resolution shown below.

3 $$\Delta v=\frac{\lambda }{2{T}_{{{{{\rm{chirp}}}}}}}$$

## Discussion

The demonstrated biaxial OPA-FPA architecture provides a system-level approach to solid-state FMCW LiDAR by combining an OPA transmitter with a coherent FPA receiver on a single silicon photonics platform. By implementing transmission and reception on separate optical paths, the architecture eliminates the need for an optical circulator, reducing insertion loss and simplifying photonic integration. At the same time, it integrates the complementary strengths of OPAs and FPAs, with the OPA enabling agile line-by-line beam steering and high optical power handling, and the FPA providing a large effective receiving aperture and parallel coherent signal acquisition at a single wavelength. Moreover, monolithic integration lithographically co-registers the OPA transmitter and FPA receiver on the same chip, reducing relative angular misalignment while simplifying packaging and providing a scalable route towards lower-cost LiDAR modules. The performance of the proposed system is validated through single-wavelength three-dimensional imaging with simultaneous range and velocity sensing, achieved using milliwatt-level emitted optical power. High frame-rate point cloud acquisition is supported by the architecture, demonstrating its suitability for dynamic scene perception. Together, these results highlight the practicality and compactness of the biaxial OPA-FPA architecture and indicate its potential as an effective system-level solution for integrated FMCW LiDAR.

From a link-budget perspective, several routes exist to extend the achievable detection range further. The SiN can be upgraded from plasma-enhanced chemical vapor deposition (PECVD) to low-pressure chemical vapor deposition (LPCVD), yielding propagation losses as low as 0.21 dB/cm at 1550 nm while maintaining compatibility with monolithic Ge/Si PDs^33,34^. For the approximately 1 cm routing length employed here, this improvement corresponds to a reduction of around 3 dB in waveguide loss. In addition, the line-shaped far-field beam emitted by the OPA is broader than the effective collecting area of the receiving gratings (Supplementary Fig. 4), leading to a fill-factor loss of approximately 12 dB that is primarily determined by the receiver pixel pitch. Furthermore, each pixel employs a periodic grating receiver with a numerical aperture (NA) of about 0.18, which is smaller than the lens numerical aperture of 0.35, resulting in an additional approximately 6 dB loss due to angular mismatch. These losses, together with the simulated approximately 3 dB mode-mismatch loss between the lens-focused field and the grating receiver, could be mitigated by high-NA grating designs^35^ and integrating microlens^19,36^ and metalens^37^ arrays to enhance angular acceptance.

The spatial resolution of FPA-based receivers is fundamentally constrained by the receiver pixel pitch. Prior studies have demonstrated substantially shorter PD, with length down to 4 μm while maintaining responsivities around 0.8 A/W^38^. Similarly, the directional coupler used for coherent mixing could be reduced from 20 μm to approximately 10 μm by migrating from a 200-mm to a 300-mm fabrication platform enabled by immersion lithography with a resolution of 60 nm^39^. In addition, the footprint of the grating receivers can be reduced to a 3 μm scale^8^. These component-level optimizations suggest the feasibility of scaling the receiver pixel pitch from the present 75 μm to approximately 20 μm with customized designs, enabling higher instantaneous spatial sampling density without altering the underlying system architecture.

Moreover, integrated laser sources represent a promising pathway toward fully integrated and scalable FMCW LiDAR systems. Direct frequency chirping through electrical modulation of the laser drive is particularly well suited to integrated lasers, offering advantages in compactness and link-budget efficiency over external modulation schemes such as in-phase and quadrature (IQ) modulation. With established techniques for chirp linearization, fully on-chip FMCW light sources become feasible, and recent demonstrations indicate that integrated lasers with output powers reaching hundreds of milliwatts are achievable^40^. Together with the OPA-FPA architecture demonstrated here, these developments point toward compact, high-performance, and scalable solid-state LiDAR systems for next-generation intelligent sensing applications.

Finally, beyond photonic integration, the demonstrated architecture is also compatible with emerging approaches in optoelectronic co-integration. In particular, electronic-photonic integrated circuits (EPICs) and co-packaged optics (CPO) provide viable pathways to further reduce system footprint and interconnect complexity by bringing high-speed electronic control and signal processing into close proximity with the photonic chip^9,41^. In the present implementation, electronic driving, readout, and data processing are realized off chip. Future implementations could leverage heterogeneous integration or advanced packaging to co-integrate photonic transmit-receive arrays with electronic drivers, analog front ends, and digital processing units. Such integration would reduce parasitic losses, relax bandwidth constraints at electrical interfaces, and enable more compact and energy-efficient LiDAR modules. Importantly, the separation of optical beam steering and coherent reception in the OPA-FPA architecture naturally aligns with these hybrid integration strategies, as the photonic and electronic subsystems can be optimized and scaled independently.

## Methods

### Design and fabrication

The biaxial LiDAR chip was fabricated using imec’s iSiPP200 silicon photonics platform^34^. The waveguide stack was realized on a silicon-on-insulator (SOI) substrate with a 220-nm crystalline silicon device layer. A 160-nm polycrystalline silicon layer was deposited above the silicon device layer to improve grating efficiency^42^. Germanium layers for PDs and a 400-nm PECVD SiN layer were subsequently integrated. Thermal tuning was implemented using tungsten heater layers, followed by two copper metallization layers for electrical routing and matrix addressing^28^. All dies were characterized at wafer-level using an automated measurement setup. At a wavelength of 1550 nm, the average propagation losses were measured to be 0.5 dB/cm for 1-μm-wide multimode silicon waveguides and 3.5 dB/cm for single-mode PECVD SiN waveguides, which were primarily used for waveguide routing.

Most photonic components were implemented using imec’s standard process design kit (PDK). Custom-designed components included low-power thermo-optic phase shifters, phase interrogators, grating antennas for the OPA transmitter, and grating receivers for the FPA. The circuit layout was designed using Luceda IPKISS and Cadence Virtuoso, and design-rule checking was performed using Mentor Graphics Caliber.

### Prototype packaging

The photonic chip was mounted on a copper heat spreader through a cutout in the PCB, enabling electrical interconnection via wire bonding and edge coupling to a fiber array. A thermoelectric cooler (TEC) and heat sink were positioned beneath the copper heat spreader. Thermal control was implemented using a TEC controller (TEC-1091) and two thermistors located in close proximity to the photonic chip and the TEC. The analog front-end electronics were placed adjacent to the photonic chip on the same PCB. Amplified differential electrical signals, together with control and drive signals, were routed via a high-speed shielded flexible cable to the RFSoC board. To reduce mechanical vibration coupling from the actively cooled electronics, the system was partitioned into separate PCBs for the photonic chip and the actively cooled components. All components, including the external FMCW laser, imaging optics, PCBs, and the thermal management unit, were integrated into a compact enclosure, forming a fully integrated biaxial LiDAR prototype.

### Experimental devices

The commercial continuous-wave laser (Thorlabs DFB1550P, with a measured linewidth of 200 kHz at a drive current of 150 mA) was driven by a laser controller (Koheron CTL200 CB-600). The generated FMCW signal was amplified using an erbium-doped fiber amplifier (Thorlabs EDFA300P) and subsequently coupled into the photonic chip. For 3D imaging over a large FOV, a compact imaging lens (EHD0814H1SW, *f* = 8 mm, *f*/1.4) was employed, whereas a 1-inch imaging lens (EHDNV3514SW, *f* = 35 mm, *f*/1.4) was used for the long-range measurements. System-level control was provided by an RFSoC (Xilinx Zynq Ultrascale + RFSoC ZU29/39/49DR SOM). Parallel signal readout was achieved using high-speed TIAs (OPA857IRGTT), followed by an anti-aliasing filter and analog-to-digital converters (ADCs).

### Chirp linearization

The frequency tuning of the commercial continuous-wave laser exhibits a nonlinear dependence on the applied drive signal. As a result, chirp calibration and linearization are required to generate a linear FMCW waveform^43^. The experimental setup used for chirp linearization is shown in Supplementary Fig. 6a. A fiber delay line with a length of 35 m was employed to emulate the round-trip propagation delay corresponding to a 25 m free-space ranging distance. An arbitrary waveform generator (AWG, Tektronix AFG3252) provided a linear chirp waveform to the laser driver, producing a maximum frequency excursion of 3 GHz. The same AWG simultaneously supplied a trigger signal to the oscilloscope (Keysight MSOX3024A) to ensure synchronization. A variable optical attenuator (VOA) was used to adjust the optical power and prevent saturation or damage of the discrete BPD (Thorlabs PDB570C). The resulting beat signal was detected by the BPD and recorded by the oscilloscope. Digital waveforms from both the AWG and the oscilloscope were transferred to a computer for offline processing. Chirp linearization was performed through an iterative calibration procedure, in which the instantaneous phase of the measured beat signal was extracted using a Hilbert transform and compared with that of an ideal linear chirp generated by the AWG. Based on the measured frequency error, the drive waveform was updated iteratively. The iteration index is denoted by *i*, and the total number of iterations, *N*_*loop*_, was manually set. As shown in Supplementary Fig. 6b, after four iterations, the maximum frequency deviation was reduced from 250 MHz to 1.5 MHz over a 3 GHz sweep, corresponding to a normalized frequency error of 0.05%. The linearized chirp is shown as the red curve in Fig. 3b.

### Sigma-delta modulation

High-resolution phase control in the OPA transmitter and the MZI-tree was implemented using sigma-delta modulation with a calculated root-mean-square (RMS) phase noise of approximately 3° per phase shifter^44^. In this scheme, the desired analog phase command was encoded into a high-speed, low-bit digital bitstream generated by a sigma-delta modulator. Instead of applying a static multi-level voltage, the phase shifters were driven by a binary voltage sequence switching between discrete levels, typically 0 and *V*_*max*_. Over a modulation period that is significantly shorter than the thermal or electrical response time of the phase shifter, the device effectively experiences an average voltage determined by the duty cycle of the bit stream. The sigma-delta modulator continuously shapes the quantization error, pushing it to high frequencies outside the signal bandwidth. The intrinsic low-pass response of the phase shifter suppresses the high-frequency switching components, resulting in a smooth and accurate effective drive voltage and, consequently, precise phase control. This pulse-density-based approach enables fine phase resolution while relaxing the requirements on multi-bit digital-to-analog converters. Moreover, the use of simple digital drivers reduces wiring complexity and improves scalability, which is particularly advantageous for large-scale OPA and MZI arrays.

### BPD characterization

Compared to a conventional BPD architecture, two additional silicon diodes were integrated to enable cross-point addressing and suppress inter-pixel crosstalk. The PDs in the FPA receiver exhibit a responsivity of approximately 1.1 A/W under a reverse bias of  −1.65 V, with a dark current of 27 nA at 1550 nm (Supplementary Fig. 8a–c)^45^. The common-mode rejection ratio (CMRR) of the BPD quantifies its ability to suppress signals that are identical at both PDs, such as laser intensity noise and background fluctuations. A higher CMRR more effectively cancels these common-mode contributions, thereby improving the signal-to-noise ratio and enabling shot-noise-limited coherent detection. The experimental setups for CMRR characterization are shown in Supplementary Fig. 8d, e. It is defined as:

4 $${{{{\rm{CMRR}}}}}({V}_{{{{{\rm{b}}}}}})=20 \, {\log }_{10}\left(\frac{\left\vert {I}_{{{{{\rm{b}}}}}}\right\vert }{\left\vert {I}_{1}\right\vert+\left\vert {I}_{2}\right\vert }\right)$$

where *I*_1_ and *I*_2_ denote the measured photocurrents when only the left or right PD is active, corresponding to the “ON" state with *V*_b_ < 0 and the “OFF" state with *V*_b_ > 0, respectively, and *I*_b_ represents the photocurrent of the BPD when both PDs are operated simultaneously under reverse bias ("ON” state, 0 < *V*_b_ < 3.3 V). Under a reverse bias of 1.65 V, the inter-pixel crosstalk and the CMRR of the BPD are measured to be  − 30 dB and 42 dB, respectively (Supplementary Fig. 8f, g).

## Supplementary information

Supplementary Information

Transparent Peer Review file

## Data Availability

All data supporting this study are available in the paper and Supplementary Information. Additional data related to this paper are available from the corresponding authors upon request.
