# Supplementary material for: Monolithically integrated silicon photonic biaxial FMCW LiDAR system
Source: Nat Commun. 2026 Sep 4;17:10538. doi: 10.1038/s41467-026-77490-4 (PMC13642332; doi:10.1038/s41467-026-77490-4)
Supplement: Supplementary file 1 [file 41467_2026_77490_MOESM1_ESM.pdf]

# Supplementary information for "Monolithically integrated silicon photonic biaxial FMCW LiDAR system"

Mathias Prost[1], Huaqing Qiu[1*], Guillaume Croes[1*], Manuel Reza[1], Jac Romme[2], Rainer Hornung[2], Javier Pérez Santacruz[1,2], Elbert Bechthum[2], Ziduo Lin[1], Jochem Govers[2], Pawel Bembnowicz[2], Brecht Berteloot[1], Esteban Venialgo[2], Erik Emmen[1], Felipe Pacheco de Oliveira[2], Shahzad Muzaffar[2], Evgenii Tiurin[2], Paul Mateman[2], Nicolas Chauvet[2], Peter Girouard[2], Tangla D. Kongnyuy[1], Mennatallah Kandil[1], Maliheh Ramezani[1], Puvendren Subramaniam[1], Padraic E. Morrissey[3], Sean Collins[3], Matthew L. Hall[3], Peter O'Brien[3], Noor Schilder[2], Christian Bachmann[2], Joost Brouckaert[1], Ruud Oldenbeuving[2], Dongjae Shin[2], Roelof Jansen[1], Peter Gerets[1], Marcus Dahlem[1]

[1]imec, Kapeldreef 75, 3001 Leuven, Belgium.
[2]imec Netherlands, HTC31 5656 AE, Eindhoven, Netherlands.
[3]Tyndall National Institute, Cork, T12 R5CP, Ireland.

*Corresponding author(s). E-mail(s): Huaqing.Qiu@imec.be; Guillaume.Croes@imec.be;

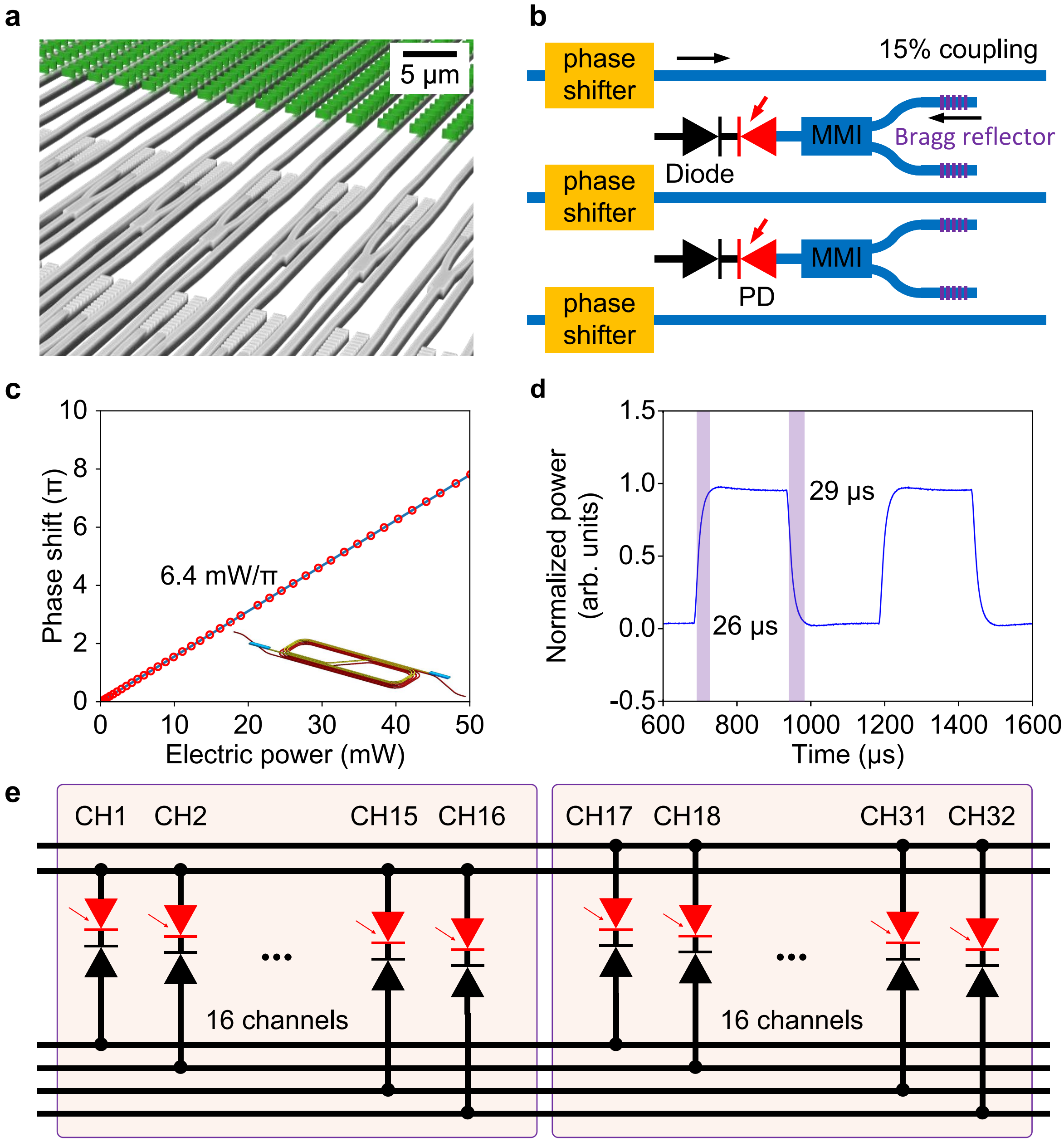


**Supplementary Fig. 1**: Design and characterization of the OPA transmitter. **a**, Schematic illustration of the short-grating emitter and the on-chip rapid phase interrogators. **b**, Design of the phase interrogator [1]. **c**, Measured power consumption of the thermo-optic phase shifter in the OPA transmitter, showing 6.4 mW/$\pi$ tuning efficiency. **d**, Measured rise and fall times of the phase shifter, corresponding to an OPA beam-steering modulation bandwidth of 12.5 kHz. **e**, The 128 OPA channels are divided into 8 blocks of 16 channels. Cross-point addressing reduces the number of bus metal wires from 256 ($128 \times 2$), required for independent phase-interrogator control, to 24 ($16 + 8$). Integrated Si diodes provide electrical isolation by suppressing sneak-current paths and dark-current leakage from unselected photodiodes, including neighbouring channels such as CH2 and CH17 that would otherwise couple into CH1, thereby mitigating inter-channel crosstalk. MMI, multimode interference; PD, photodetector. Panels **a-c** are reprinted with permission from IEEE Proceedings.

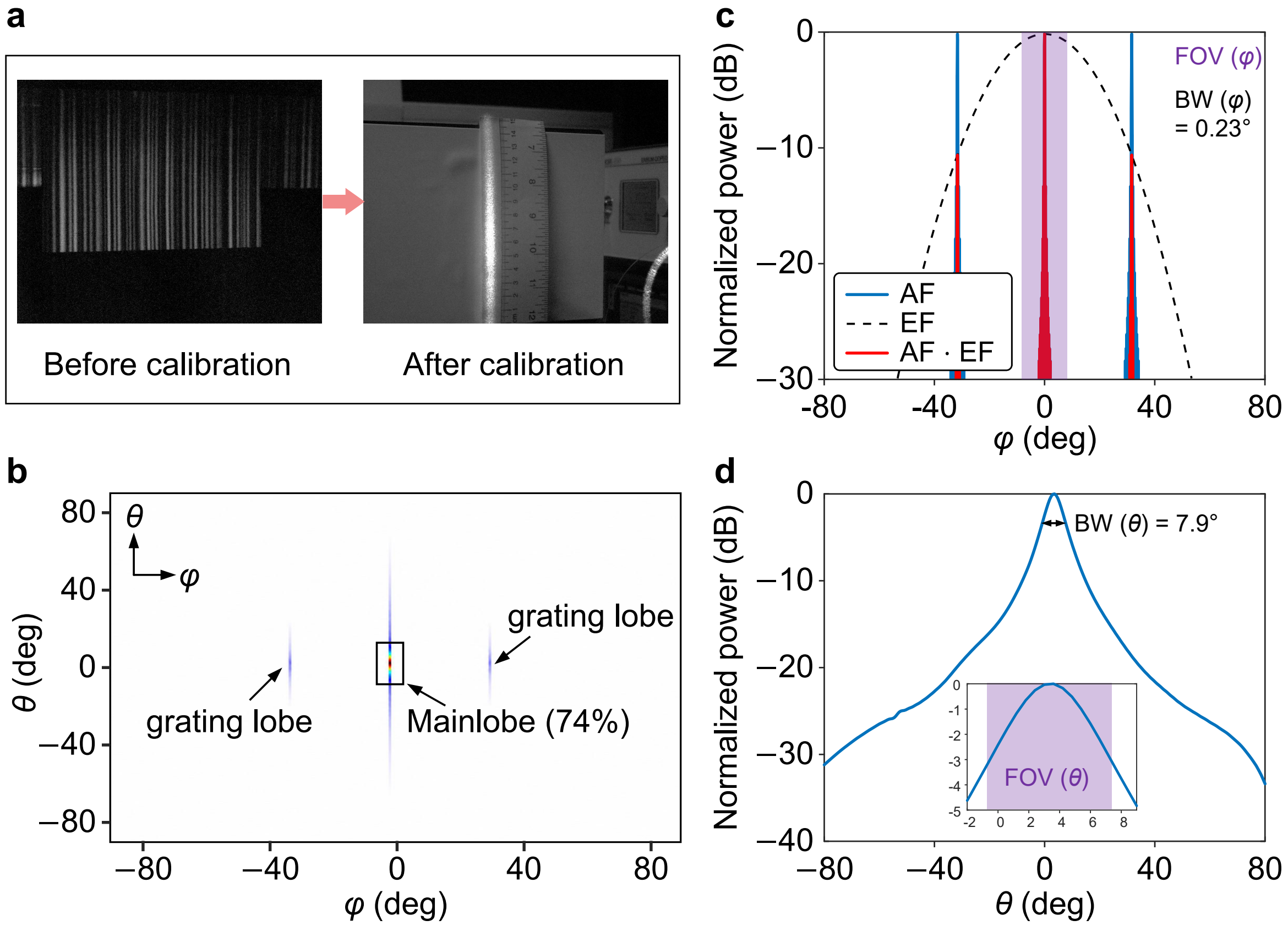


**Supplementary Fig. 2**: Calibration and simulation of the 128-channel OPA transmitter. **a**, OPA transmitter with a line-shape far-field pattern before and after calibration. **b**, Simulated far-field profile of the 128-channel OPA transmitter. The grating has a width of 2.7 µm, a designed length of 50 µm and an effective radiating length of 10 µm; the array pitch is 2.94 µm. The mainlobe 3 dB beamwidth, extracted from the boxed region, is $0.23° \times 7.9°$. **c**, Simulated and calculated OPA far-field profile in the $\varphi$ direction at $\theta = 3.3°$. The widened grating antenna narrows the element factor (EF), enabling approximately 74% of the total optical power to be concentrated within the main lobe over the 16° scanning range (FOV ($\varphi$)) of the LiDAR prototype. **d**, Simulated and calculated OPA far-field profiles in the $\theta$ direction at $\varphi = 0°$. The 10 µm effective grating length yields a $\theta$-direction beamwidth of 7.9°, matching the 8° FOV in the $\theta$ direction (FOV ($\theta$)) of the prototype. FOV, field of view, BW, beamwidth.

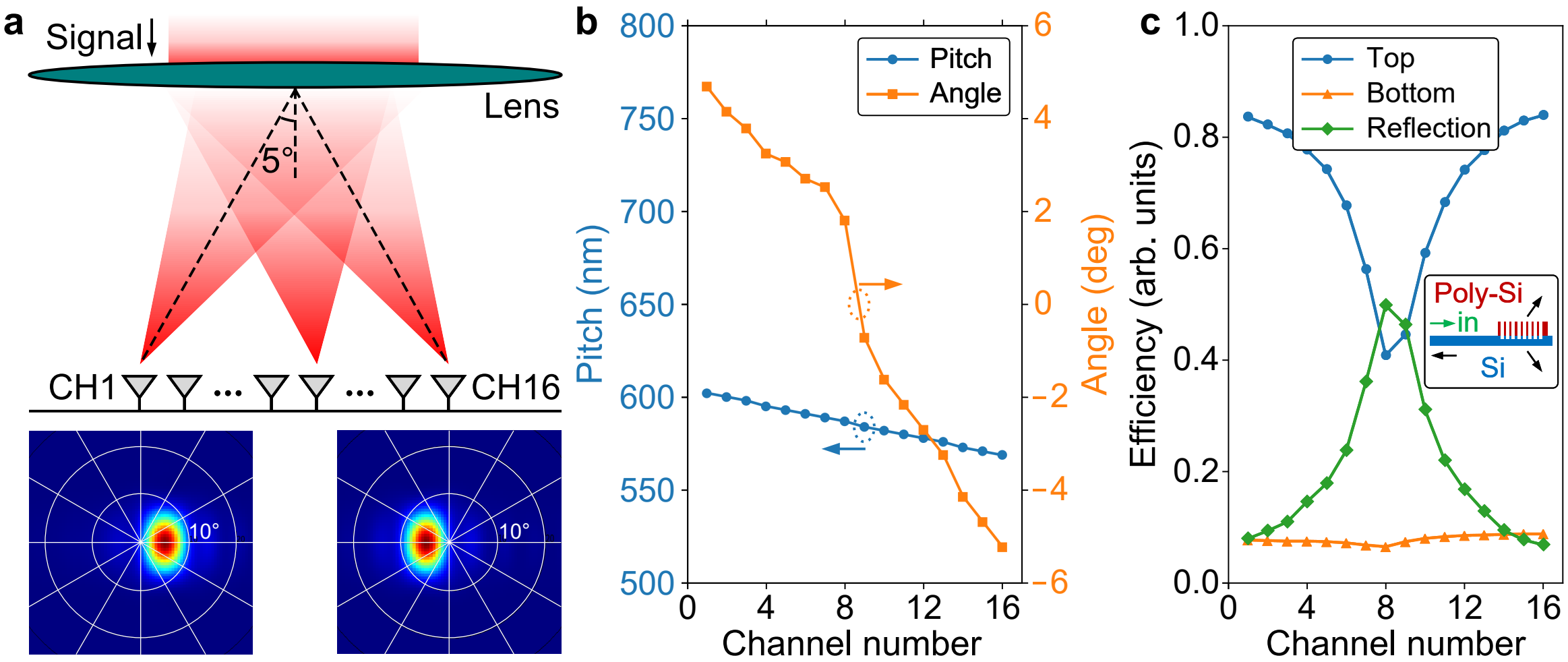


**Supplementary Fig. 3**: Design and characterization of the FPA receiver. **a**, Schematic illustration of the FPA receiver. By design, the grating receiver angles span from −4° to 4° to match the 8° FOV and ensure alignment of all gratings with the lens optical axis. Simulations based on the as-fabricated dimensions indicate an actual angular range of approximately −5° to 5°; however, this deviation introduces only a minor additional optical loss. **b**, Design of the grating pitch and corresponding simulated receiving angle for different channels. **c**, Simulated grating coupling efficiency and back-reflection for different receiver channels. Although the central channels with grating emission angles of ±1.5° exhibit high back-reflection, this does not affect their operation as receivers for free-space coupling into the photonic chip. Si, silicon; SiN, silicon nitride.

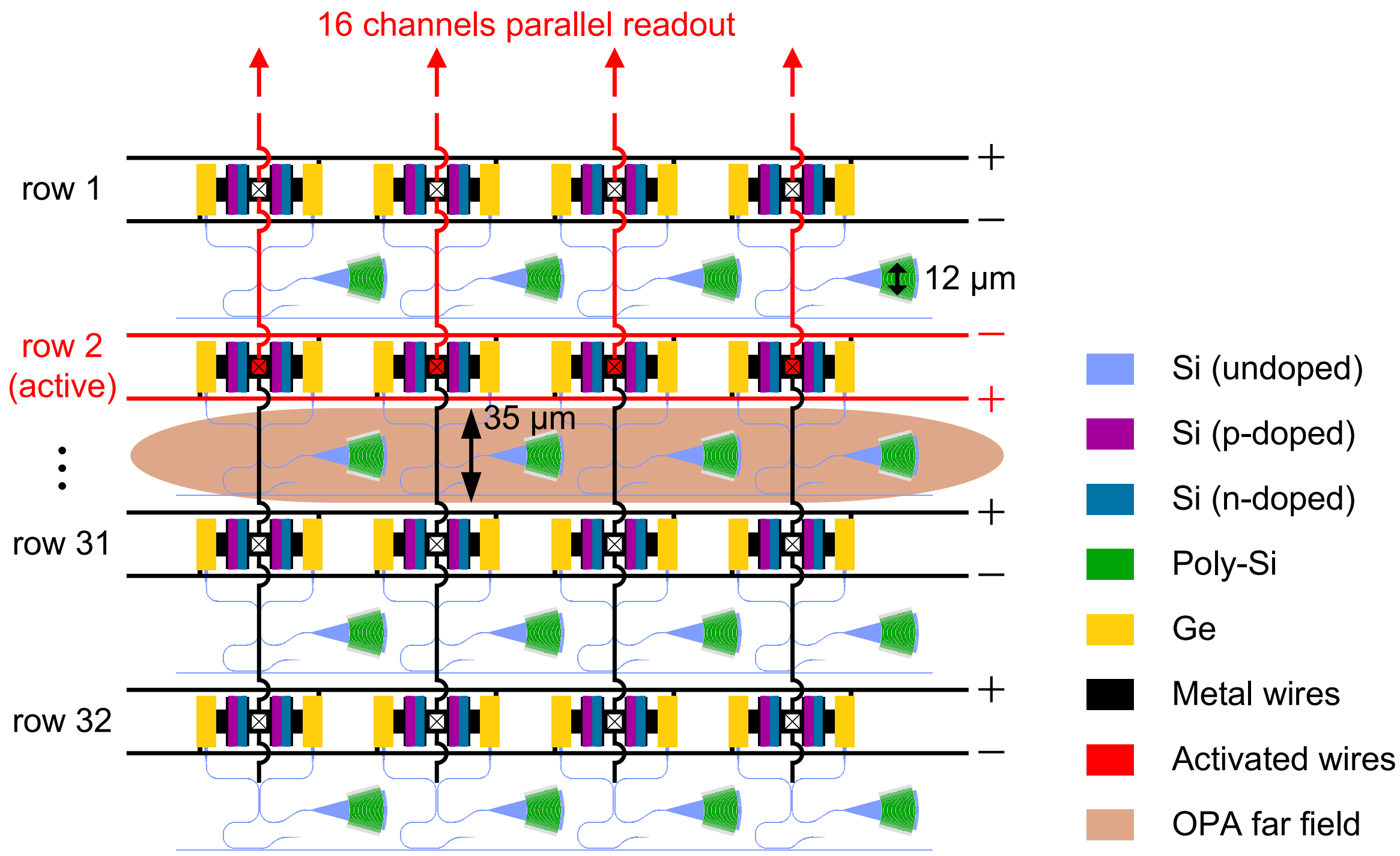


**Supplementary Fig. 4**: Schematic of FPA parallel readout. The 16 BPDs in the selected row are reverse-biased and activated for parallel readout of 16 differential photocurrents. Si diodes integrated between the two PDs in each BPD suppress inter-channel and inter-pixel crosstalk, enabling cross-point addressing that reduces the bond-pad count from $N^2$ to $N$-level scaling. The received beam, generated by OPA emission, target reflection and imaging through an 8-mm-focal-length lens ($f/1.4$), forms a 35 μm × 1.2 mm footprint on the image plane. Its 1.2-mm length spans a full pixel-array row, while its 35-μm width covers the receiving gratings, which have an effective collection area of approximately 12 μm × 15 μm, resulting in a fill-factor loss of approximately 12 dB. Si, silicon; Ge, germanium; OPA, optical phased array.

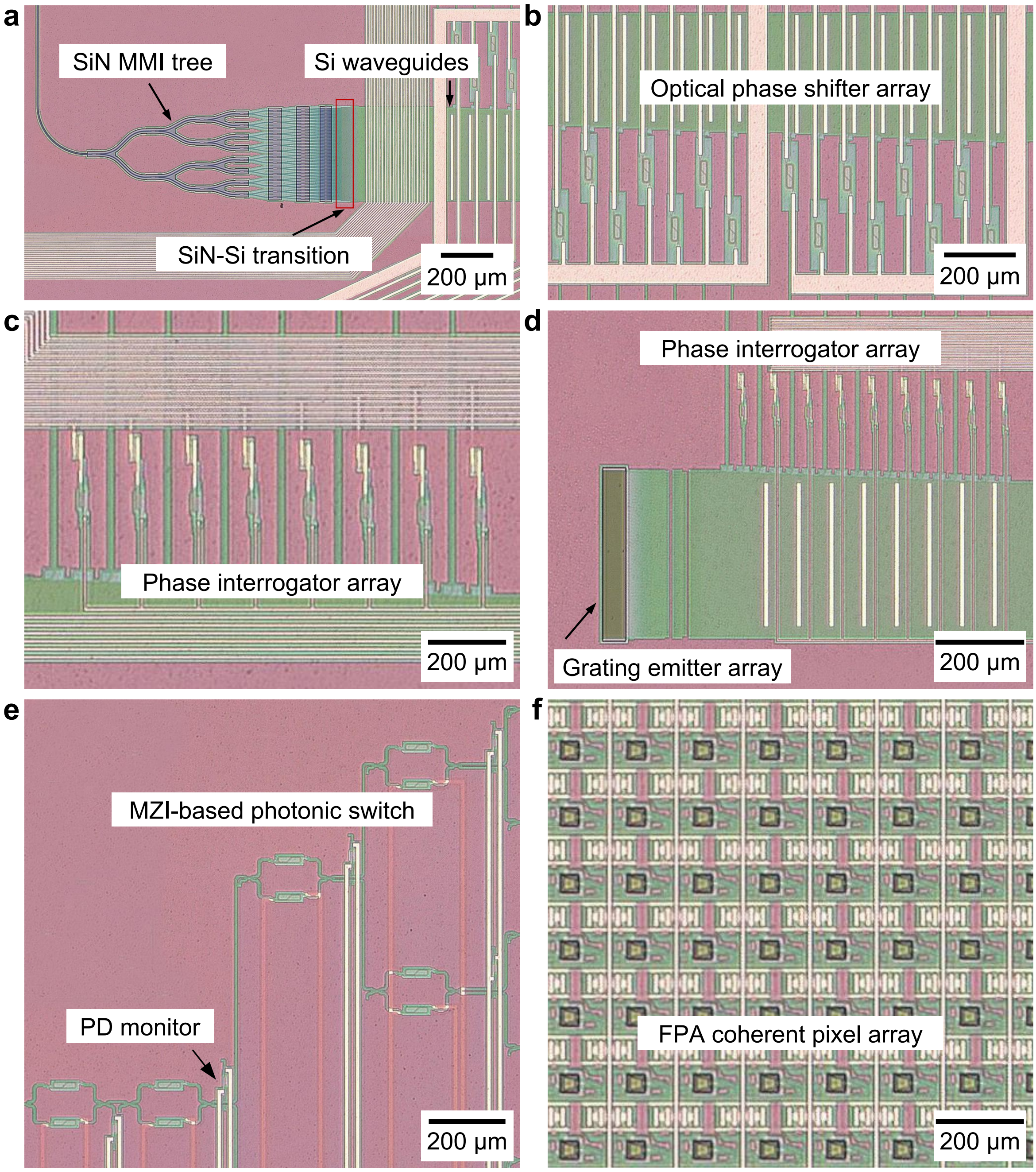


**Supplementary Fig. 5**: Microscope images of the biaxial LiDAR components. **a**, SiN MMI tree fabricated using PECVD SiN, distributing optical power from a SiN bus waveguide into the 128-channel waveguides. The SiN bus waveguide sustains an optical power of 60 mW in the experiment, which is evenly split into 128 SiN waveguides and subsequently converted to Si waveguides for energy-efficient phase tuning through SiN-Si transitions, enabling high-power operation of the OPA. **b**, Energy-efficient optical phase-shifter array. **c**, Phase-interrogator array used for rapid on-chip calibration, requiring 1.4 s per channel with 7 repeated measurements and averaging to enhance robustness. **d**, Short grating emitters forming the line-shaped far-field of the OPA transmitter. **e**, Reconfigurable MZI-based photonic switch array, where each MZI integrates a PD monitor for ON/OFF state verification. **f**, 2D coherent pixel array of the FPA receiver. Si, silicon; SiN, silicon nitride, MMI, multimode interference; PD, photodetector, MZI, Mach–Zehnder interferometer; FPA, focal plane array.

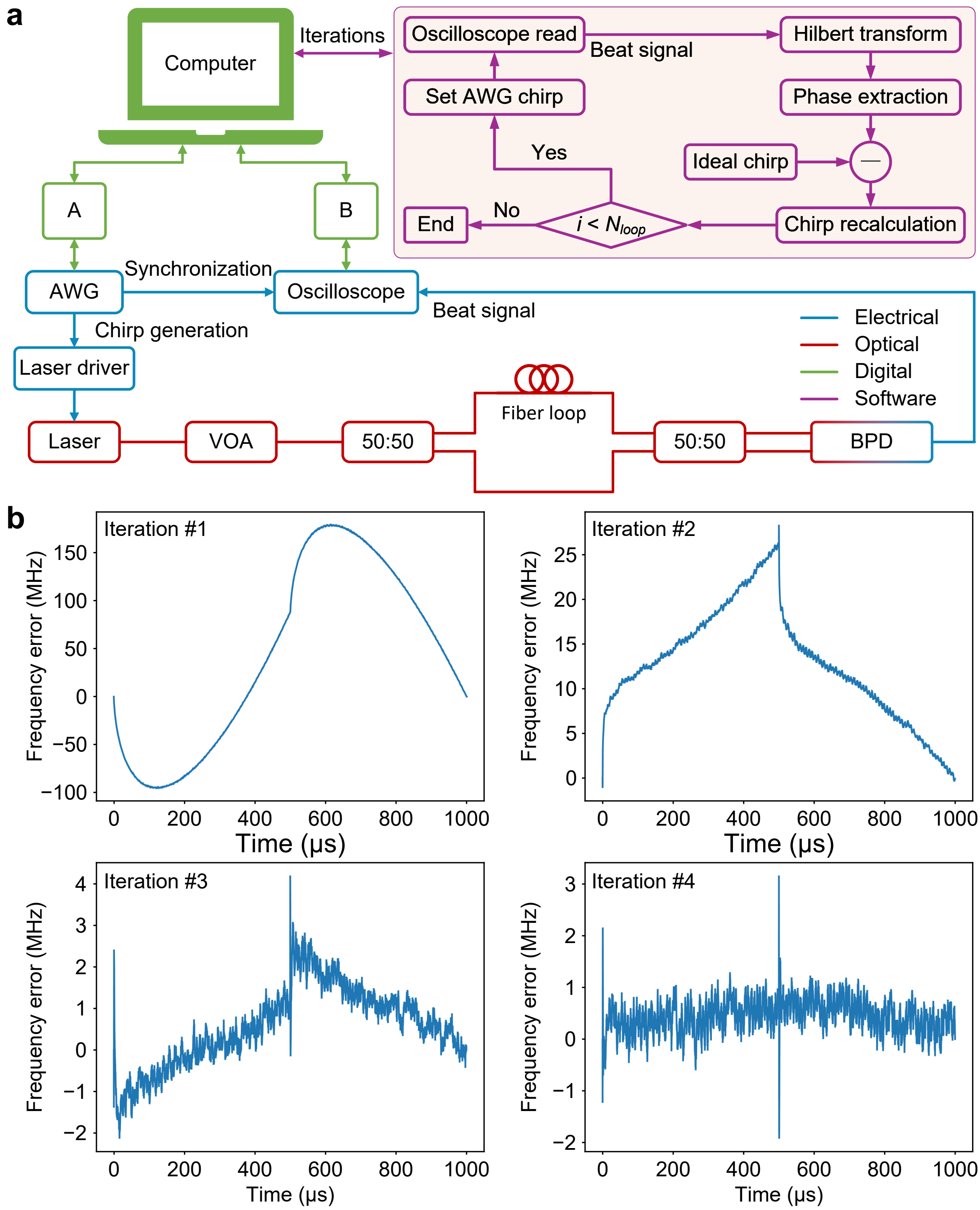


**Supplementary Fig. 6**: Chirp linearization schematic and characterization. **a**, Schematic of chirp linearization for a directly modulated FMCW laser. A 35 m fiber loop is used to emulate the round-trip propagation delay of a target located at 25 m in air. An AWG generates the triangular chirp waveform and provides the trigger signal for oscilloscope synchronization. The Hilbert transform is applied to extract the instantaneous phase of the beat signal, which is compared with the ideal triangular chirp waveform generated by the AWG to evaluate the frequency error. The index $i$ denotes the iteration number, and $N_{loop}$ represents the total number of iterations. VOA, variable optical attenuator. **b**, Measured frequency error over the chirp duration versus iteration number. Each 1000 µs triangular chirp waveform is sampled with 4096 points. After four iterations, the maximum frequency deviation is reduced from 250 MHz to 1.5 MHz over a 3 GHz sweep, corresponding to a normalized frequency error of 0.05%. AWG, arbitrary waveform generator; BPD, balanced photodetector; VOA, variable optical attenuator.

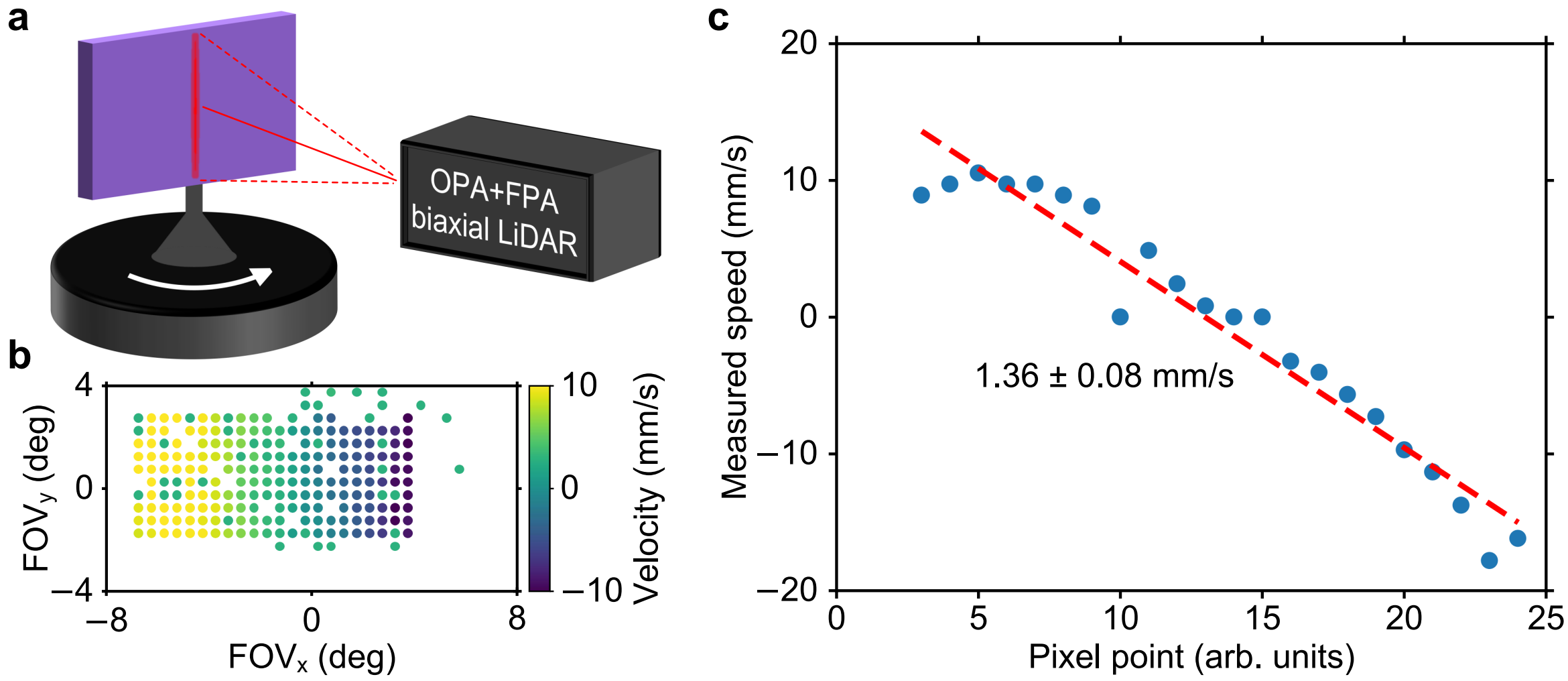


**Supplementary Fig. 7**: Velocity resolution measurement. **a**, Experimental setup of a rectangular board of 20 cm width mounted on a rotating stage at 1 revolution per minute (rpm). The LiDAR prototype was positioned 1.1 m away. **b**, The Velocity point cloud measured from the rectangular board. **c**, The measured velocity resolution is $1.36 \pm 0.08$ mm/s, in reasonable agreement with the theoretical value of approximately 0.8 mm/s. OPA, optical phased array; FPA, focal plane array; FOV, field of view.

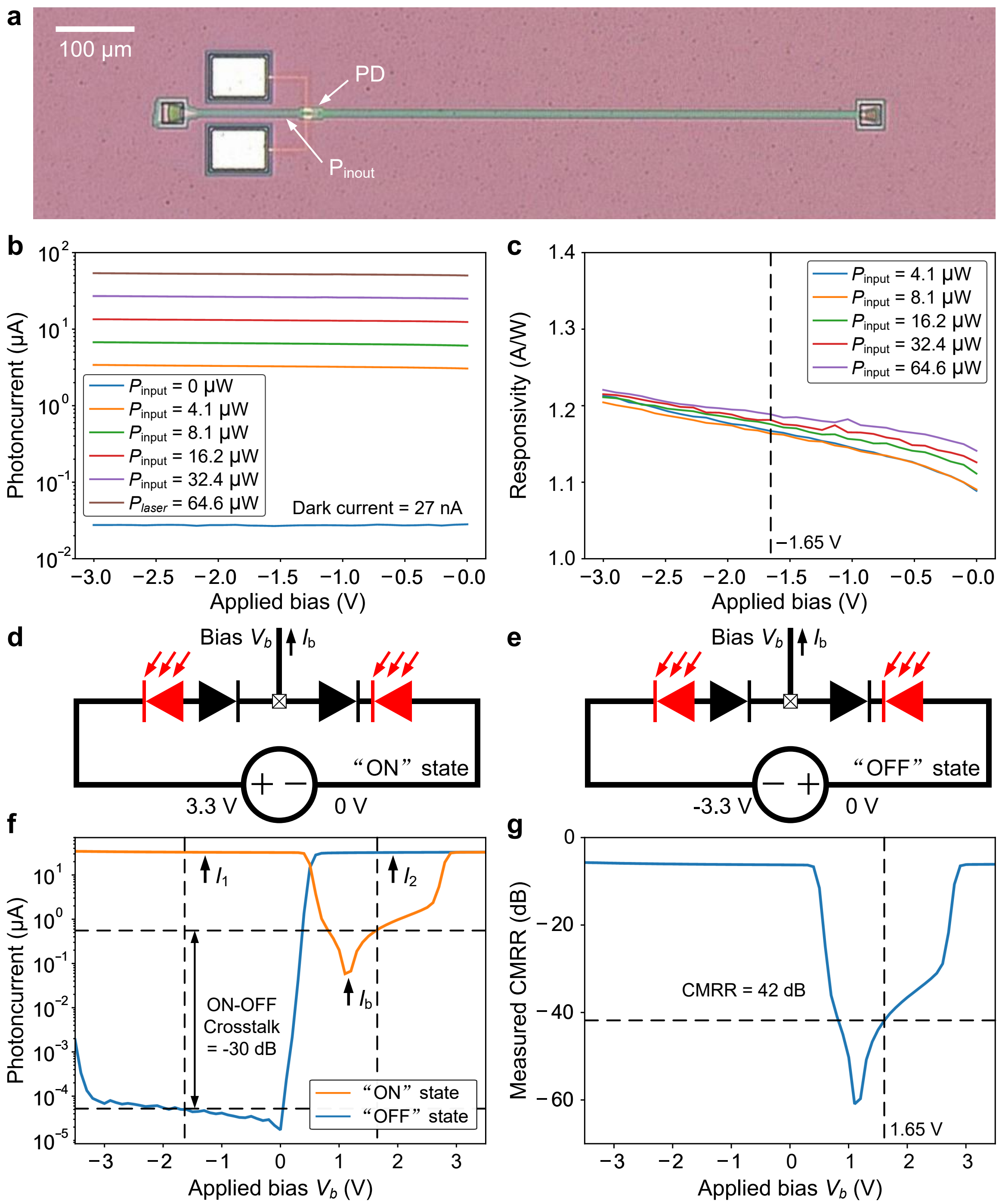


**Supplementary Fig. 8**: PD characterization at 1550 nm. **a**, Optical microscope image of the PD test structure. **b,c**, Measured photocurrent and responsivity as functions of incident optical power, indicating a dark current of 27 nA and a responsivity of approximately 1.1 A/W at a reverse bias of −1.65 V. **d,e**, Measurement schematics for CMRR and inter-row crosstalk characterization of the BPD under reverse-biased ("ON") and forward-biased ("OFF") operation. The applied voltages are 3.3 V/0 V for the "ON" state and −3.3 V/0 V for the "OFF" state, while the differential bias between the two PDs is swept from −3.3 V to 3.3 V. **f**, Measured BPD photocurrent responses under the "ON" and "OFF" states, indicating an inter-row crosstalk level of −30 dB. **g**, Measured CMRR of the BPD, reaching up to 42 dB, indicating good fabrication consistency of the PDs and silicon diodes. PD, photodetector; CMRR, common-mode rejection ratio.

# References

[1] Prost, M. *et al.* *Silicon photonic fmcw lidar with integrated high-speed line-scan illumination and 2D coherent receivers*, 1–4 (IEEE, 2025).
