# Supplementary material for: Monolithically integrated silicon photonic biaxial FMCW LiDAR system
Source: Nat Commun. 2026 Sep 4;17:10538. doi: 10.1038/s41467-026-77490-4 (PMC13642332; doi:10.1038/s41467-026-77490-4)
Supplement: Supplementary file 2 [file 41467_2026_77490_MOESM2_ESM.pdf]

nature portfolio

Peer Review File

Monolithically integrated silicon photonic biaxial FMCW LiDAR system

Corresponding Author: Dr Huaqing Qiu

This file contains all reviewer reports in order by version, followed by all author rebuttals in order by version.

Version 0:

Reviewer comments:

Reviewer #1

(Remarks to the Author)
The authors demonstrated a biaxial/bistatic FMCW LiDAR prototype using an optical phased array (OPA) as a transmitter and a focal plane array (FPA) as a receiver. This complementary architecture combines agile beam steering of OPAs with the parallel detection capabilities of FPAs. The methods and results are informative and comprehensive, and they represent a major step toward practical application, given the effort involved. I would advocate for its publication in Nature Communications if the following concerns were addressed.

1. The authors claimed that they engineered a line-shaped beam for their OPA. Except for the SWIR image in Extended Data Fig. 1 (e), there are no methods or results concerning the engineering or characteristics of this line shape. For example, did they achieve a uniform intensity distribution along the line? They also mentioned that the line is broader than the effective collecting area of the receiving gratings, resulting in a 12 dB fill-factor loss. Can they elaborate on this issue? For example, how is the loss evaluated?
2. In addition to the fill-factor-induced loss, another major issue in adopting an OPA-FPA hybrid architecture is the field-of-view (FOV) limitation imposed by the RX lens. Can the authors comment on how this issue can be alleviated in the future, provided that good coupling to individual RX gratings is maintained? In other words, can they comment on how they plan to extend their 16° × 8° FOV in the future?
3. Judging from the title and the manuscript, one of the key contributions appears to be monolithic integration. Can they comment on whether the monolithic integration of both OPA and FPA on a single chip yields substantial benefits? For example, does the monolithic integration reduce directional/angular misalignment at closer ranges, since they used a bistatic setup?
4. The authors claimed in lines 127 & 128 that the on-chip phase calibration time is limited by the read-out capability and can be improved to 2.5 ms per channel. Can they support this claim with more details? For example, how long does it take to read the photon current and feed it back to the TO phase shifter? How many steps does it take to tune one channel? And how is it reduced to 2.5 ms? Additionally, is 2.5 ms fast enough in the future? Will it limit their framerate if a finer resolution is required? As a side note, in the caption of Extended Data Fig. 4, they reported a calibration time of 1.4 s per channel. Please clarify this inconsistency.
5. Similarly, it is desirable to provide a detailed time breakdown of the reported frame rate, comparing potential limiting factors, including OPA direction-to-direction scanning time, transceiving time, laser chirp rate, electronics configuration, read-out time, and processing time. Only by specifying each limiting factor can the authors support their claim that a 62 fps frame rate is potentially achievable.
6. In line 146, the authors wrote that the BPD includes two silicon diodes for cross-point addressing. Can they elaborate on the functionality of these two diodes and the diode attached to the phase interrogation PD in their OPA? It appears that diodes are used to enable and disable PDs with DC biasing, but the current manuscript is not explicit enough.
7. In lines 148 and 149, the authors wrote that the receiving angles vary across channels but are identical across rows within each channel. Can they elaborate on why the receiving angles are identical across rows within each channel? Is this a physical fact or a design limitation? Ideally, the diffraction from each grating should point toward the lens center, resulting in different receiving angles across the array.
8. Following up on comment 7, can the authors elaborate on how they map the receiving angular range of 5° to -5° to their 16° × 8° FOV? Is this determined by their lens design? Additionally, did they implement any measures to reduce inter-pixel crosstalk within the same row? For example, is there observable crosstalk between the central grating for near-normal receiving and its neighbors?

9. Can the authors comment on the total power consumption of their prototype, ideally breaking down into each sub-assembly, including the laser, OPA, FPA, RFSoC, and TECs?
10. The authors claim that their sigma-delta modulation (SDM) achieved precise phase control without giving the equivalent phase/bit resolution. Please support your claim quantitatively.
11. In lines 172 and 173, the authors wrote that the signals are then anti-aliasing filtered. Can they elaborate on this procedure and its purpose?
12. Can the authors comment on how many points are stored in their 4 kB ADC register for a 500 μs chirp, and how they determine the chirp flip point (i.e., from up-chirp to down-chirp or vice versa) in their stored data?
13. It appears that they are measuring targets coated with reflective tape. They reported a reflectivity of ~70% at 1550 nm. Can they elaborate on how this reflectivity is measured?
14. In Extended Data Fig. 5, the linearization is performed with a chirp rate of 1 kHz (1 ms), whereas the chirp signal shown in Fig. 3(b) has a duration of 500 μs. Please clarify whether two chirp rates are used or whether the ADC captures only part of the chirp.

Reviewer #2

(Remarks to the Author)
This manuscript presents a highly innovative, monolithically integrated biaxial FMCW LiDAR system, cleverly combining an OPA transmitter and a coherent FPA receiver—comprising over 4,500 components—on a single silicon photonic chip. This biaxial architecture successfully circumvents the reliance on on-chip optical circulators and achieves 3D imaging at a single wavelength without broadband tuning, marking an impressive step forward in the chip-level integration of solid-state LiDAR. The prototype packaging and system-level demonstrations also exhibit a high standard of engineering. However, to meet the rigorous publication standards of this journal and to genuinely substantiate the system's practical value in "dynamic scene perception," the manuscript contains several critical flaws regarding its velocity calibration methodology, presentation of raw data, and validation of high-frame-rate imaging quality. Therefore, I recommend a Major Revision to address the following issues before a final decision can be made.
1, the methodology for calibrating the velocity resolution lacks metrological rigor. The authors utilized a "square-board target with spatially varying motion" in Extended Data Fig. 6 to claim a 1 mm/s velocity resolution, yet they failed to establish any ground truth for the physical velocities across different regions of this target. A qualitative color heat map alone cannot rule out whether these minute velocity variations stem from low-frequency phase noise, ambient mechanical vibrations, or algorithmic artifacts. I strongly advise the authors to perform quantitative testing using a known, precisely calibrated moving target (e.g., a high-precision motorized linear translation stage or a spinning disk with a known angular velocity) and to provide a statistical analysis encompassing multiple velocity steps to scientifically and irrefutably validate their velocity resolution limit.
2, the presentation of the point cloud data involves artificial modifications that obscure the system's true signal-to-noise ratio (SNR) and detection performance. When demonstrating the 20-meter 3D imaging in Fig. 4b, the authors artificially added a purple virtual background point cloud behind the target, conceding in the text that this was included solely to "enhance the visual contrast." In a rigorous hardware characterization paper, implanting non-physical, virtual data is unacceptable, as it severely misleads the reader regarding the system's true field of view, point cloud sparsity, and background noise distribution. The authors must remove all virtual point clouds in the revised manuscript and present the raw, unaltered measurement results to objectively reflect the true imaging quality without the aid of a high-contrast artificial background.
3, there is a distinct disconnect in the validation linking high frame-rate operation to actual single-frame imaging quality. Although the paper claims an experimental scanning rate of 10 fps, the evaluation of the detection probability for the target at 20 meters (Fig. 4d) relies on "100 accumulated frames." This inadvertently reveals that under the extremely short single-frame dwell time at 10 fps—restricted by the weak emitted power (3.7 mW)—the true SNR of a single scan is highly likely insufficient for effective imaging. To truly prove that the system is capable of dynamic scene perception, the authors cannot rely on static accumulation to mask poor single-frame quality. They must provide raw, single-frame dynamic point clouds captured at the nominal 10 fps continuous scanning rate, demonstrating that the system can output clear, complete, and usable point clouds without severe motion blur within a short integration time.
4, the current test scenarios and target parameters are overly idealized, failing to adequately support the claim of a "practical system-level approach." The current near-distance ranging experiment utilized a planar target with an exceptionally high reflectivity of 70%, which is a far cry from the stringent optical conditions of real-world industrial applications (e.g., autonomous driving systems encountering 10% reflectivity from dark vehicles or clothing). The authors should supplement the manuscript with an SNR evaluation and maximum range demonstration using standard low-reflectivity targets (e.g., 10%). Furthermore, it is recommended to introduce test targets with complex 3D morphologies, depth variations, and a certain degree of ambient light interference. Showcasing the actual measurement results under these conditions would far more convincingly validate the core advantages of this coherent FMCW architecture in terms of crosstalk immunity and 3D spatial resolving power.

Version 1:

Reviewer comments:

Reviewer #1

(Remarks to the Author)
Thank you for thoroughly addressing my concerns; I now advocate for the publication of this manuscript in Nature

Communications without reservations.

Reviewer #2

(Remarks to the Author)
I thank the authors for their thorough and careful responses to the concerns I raised in my previous review. I am satisfied that the authors have adequately addressed all my concerns, and I am pleased to recommend this manuscript for publication in Nature Communications.

Open Access This Peer Review File is licensed under a Creative Commons Attribution 4.0 International License, which permits use, sharing, adaptation, distribution and reproduction in any medium or format, as long as you give appropriate credit to the original author(s) and the source, provide a link to the Creative Commons license, and indicate if changes were made.
In cases where reviewers are anonymous, credit should be given to 'Anonymous Referee' and the source.
The images or other third party material in this Peer Review File are included in the article's Creative Commons license, unless indicated otherwise in a credit line to the material. If material is not included in the article's Creative Commons license and your intended use is not permitted by statutory regulation or exceeds the permitted use, you will need to obtain permission directly from the copyright holder.
To view a copy of this license, visit https://creativecommons.org/licenses/by/4.0/

Dear editor and reviewers,

We express our sincere gratitude for the thoughtful comments provided by the reviewers on our manuscript. It is evident that the reviewers are experts in the field of integrated LiDAR, and we appreciate the time they spent on reviewing the manuscript. After thorough consideration of the reviewers' comments, we have diligently made more experiments and revised the manuscript. Presented below is a comprehensive response addressing each of the reviewers' comments. Please note that the reviewers' comments are highlighted in blue, and the revised parts are highlighted in red.

Reviewer #1: The authors demonstrated a biaxial/bistatic FMCW LiDAR prototype using an optical phased array (OPA) as a transmitter and a focal plane array (FPA) as a receiver. This complementary architecture combines agile beam steering of OPAs with the parallel detection capabilities of FPAs. The methods and results are informative and comprehensive, and they represent a major step toward practical application, given the effort involved. I would advocate for its publication in Nature Communications if the following concerns were addressed.

Reply: We appreciate the comments from the reviewer to our work and the chance to address all the concerns to improve it further.

Q1: The authors claimed that they engineered a line-shaped beam for their OPA. Except for the SWIR image in Extended Data Fig. 1 (e), there are no methods or results concerning the engineering or characteristics of this line shape. For example, did they achieve a uniform intensity distribution along the line? They also mentioned that the line is broader than the effective collecting area of the receiving gratings, resulting in a 12 dB fill-factor loss. Can they elaborate on this issue? For example, how is the loss evaluated?

Reply: We thank the reviewer's comment. More detailed information is presented below.

1. **Design of the line-shaped beam**. We designed a 128-channel OPA transmitter with an array pitch of 2.94 μm. Although the grating antenna has a physical length of 50 μm, its effective radiating length is only approximately 10 μm, indicating that most of the optical power is diffracted into free space within the first 10 μm. The resulting effective near-field aperture of the OPA is therefore 376 μm × 10 μm, yielding a simulated and calculated 3-dB beamwidth of 0.25° × 7.9° in the far field. The ~8° beamwidth along the ϑ direction is designed to match the prototype FoV of 16° × 8°, where the 16° FoV in the φ direction is achieved through phase-change beam steering. In addition, the grating antenna is widened to 2.7 μm to narrow the element factor (EF), allowing approximately 74% of the optical power to be confined within the main lobe despite the presence of grating lobes. These details have been revised and added to Extended Data Fig. 2. The line-shaped beam exhibits a non-uniform intensity profile, with a maximum centre-to-edge variation of around 3dB. This is because the 7.9° 3-dB beamwidth of the OPA along the ϑ direction is designed to match the FoV, placing the FoV edge at the 3-dB point of the beam.
2. **Imaging of the line-shaped beam onto the FPA**. Based on the effective near-field aperture of 376 μm × 10 μm and the corresponding 3-dB beamwidth of 0.23° × 7.9°, the beam emitted by the OPA transmitter is reflected by the target and imaged by an 8-mm-focal-length lens (EHD0814H1SW, f/1.4) onto the image plane, where the FPA receivers are located. The resulting beam footprint on the image plane is approximately 35 μm × 1.2 mm. The 1.2-mm length spans a full pixel-array row, while

the 35-µm width is designed to ensure coverage of the receiving grating, whose effective collection area is approximately 12 µm × 15 µm. Considering the pixel pitch of 75 µm × 75 µm, the fill-factor loss is calculated as:

$$\boldsymbol{loss = 10 \cdot \log_{10}\left(\frac{12 \times 15}{35 \times 75}\right) \approx 12\ dB}$$

<u>Revision</u>: In order to address this question in the manuscript, we have revised the text and added a figure. Please check lines 152 to 159, lines 231 to 236, and Extended Data Fig.2.

Revised manuscript: line 152 to 159:

"The simulated OPA far-field pattern is shown in Extended Data Fig. 2b, with a 3-dB beamwidth of 0.23° × 7.9°, as shown in Extended Data Fig. 2c, d, respectively. In addition, widening the grating antenna to 2.7 µm suppresses the grating lobes and increases the fraction of optical power in the main lobe, thereby reducing the effective OPA loss. As shown in Extended Data Fig. 2c, approximately 74% of the emitted optical power is confined to the main lobe within the 16° working FoV of the LiDAR prototype, which is defined by the FPA size and the imaging lens."

Revised manuscript: line 231 to 236:

"The line-shaped OPA pattern is imaged onto the pixel array with a calculated footprint of 35 µm × 1.2 mm, set by the image lens, the effective near-field aperture of 376 µm × 10 µm and the 0.23° × 7.9° beamwidth. The 7.9° beamwidth matches the 8° FoV, so the 1.2-mm length precisely spans the selected row, while the 35-µm width fully covers the grating receivers, resulting in a fill-factor loss of approximately 12 dB."

Revised manuscript: Extended Data Fig.2:

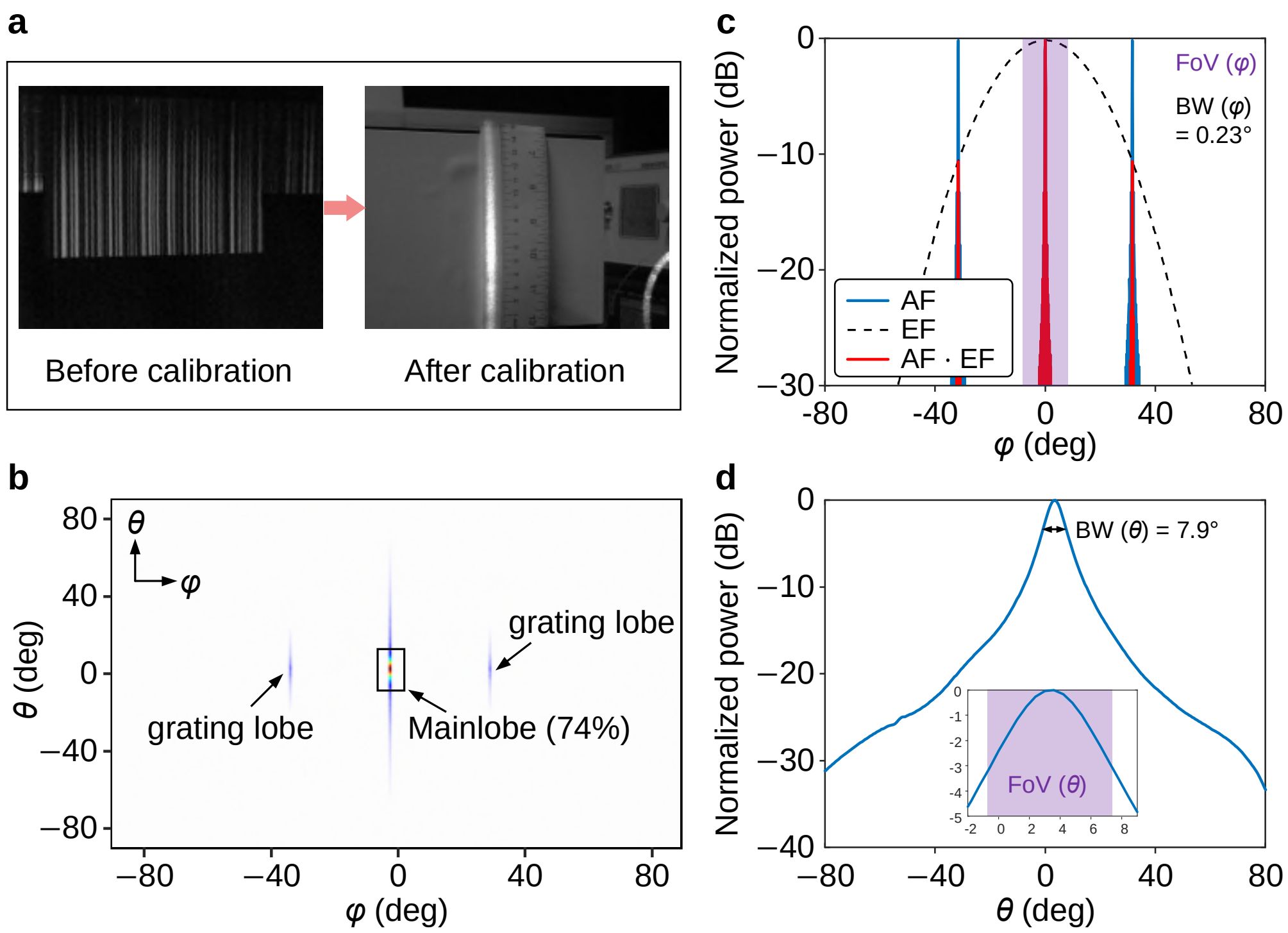

"Extended Data Fig.2: Calibration and simulation of the 128-channel OPA transmitter. a, OPA transmitter with a line-shape far-field pattern before and after calibration. b, Simulated far-field profile of the 128-channel OPA transmitter. The grating has a width of 2.7 µm, a designed length of 50 µm and an effective radiating length of 10 µm; the array pitch is 2.94 µm. The mainlobe 3-dB beamwidth, extracted from the boxed region, is 0.23° × 7.9°. c, Simulated and calculated OPA far-field profile in

the φ direction at ϑ = 3.3°. The widened grating antenna narrows the element factor (EF), enabling approximately 74% of the total optical power to be concentrated within the main lobe over the 16° scanning range (FoV (φ)) of the LiDAR prototype. d, Simulated and calculated OPA far-field profiles in the ϑ direction at φ = 0°. The 10 μm effective grating length yields a ϑ-direction beamwidth of 7.9°, matching the 8° FoV in the θ direction (FoV (ϑ)) of the prototype."

Q2: In addition to the fill-factor-induced loss, another major issue in adopting an OPA-FPA hybrid architecture is the field-of-view (FOV) limitation imposed by the RX lens. Can the authors comment on how this issue can be alleviated in the future, provided that good coupling to individual RX gratings is maintained? In other words, can they comment on how they plan to extend their 16° × 8° FOV in the future?

Reply: We thank the reviewer for this valuable comment. In the OPA-FPA hybrid architecture, the achievable field of view (FOV) is primarily determined by the physical size of the pixel array when the RX lens focal length is fixed. Therefore, one straightforward and effective way to expand the FOV, while maintaining efficient coupling to individual RX gratings, is to enlarge the pixel array.

As demonstrated in "*A large-scale microelectromechanical-systems-based silicon photonics LiDAR*" (Nature, 2022), a 70° × 70° FOV was achieved using a pixel array of approximately 7 mm together with a 5 mm focal length lens, illustrating the strong scalability of this approach. In addition, increasing the array size does not preclude high spatial resolution. Advances in silicon photonic integration enable further pixel miniaturization; for example, pixel dimensions of 8 μm × 5 μm were discussed in "*A universal 3D imaging sensor on a silicon photonics platform*" (Nature, 2021). Such scaling allows both FOV expansion and resolution improvement within a compact footprint.

Overall, enlarging the pixel array combined with continued pixel-size reduction provides a clear path toward extending the current 16° × 8° FOV while preserving good RX grating coupling efficiency.

This point has already been addressed in lines 296 to 297 of the manuscript.

Q3: Judging from the title and the manuscript, one of the key contributions appears to be monolithic integration. Can they comment on whether the monolithic integration of both OPA and FPA on a single chip yields substantial benefits? For example, does the monolithic integration reduce directional/angular misalignment at closer ranges, since they used a bistatic setup?

Reply: We thank the reviewer for the question. Although the system operates in a bistatic configuration, the monolithic integration of the OPA and FPA on a single chip substantially reduces directional and angular misalignment, particularly at shorter ranges. Because the transmitter and receiver are co-fabricated on the same substrate, their relative positions and pointing directions are defined lithographically, rather than through external mechanical or optical alignment. This chip-level co-registration minimizes relative angular uncertainty compared with discrete implementations, where small assembly tolerances can lead to noticeable near-field misalignment. While monolithic integration does not eliminate the bistatic geometry itself, it effectively mitigates practical alignment errors and improves near-range pointing consistency.

Revision: To address this question, we have revised the text in the manuscript. Please check lines 270 to 273.

Revised manuscript: line 270 to 273:

“Moreover, monolithic integration lithographically co-registers the OPA transmitter and FPA receiver on the same chip, reducing relative angular misalignment while simplifying packaging and providing a scalable route towards lower-cost LiDAR modules.”

Q4: The authors claimed in lines 127 & 128 that the on-chip phase calibration time is limited by the read-out capability and can be improved to 2.5 ms per channel. Can they support this claim with more details? For example, how long does it take to read the photon current and feed it back to the TO phase shifter? How many steps does it take to tune one channel? And how is it reduced to 2.5 ms? Additionally, is 2.5 ms fast enough in the future? Will it limit their framerate if a finer resolution is required? As a side note, in the caption of Extended Data Fig. 4, they reported a calibration time of 1.4 s per channel. Please clarify this inconsistency.

Reply: We thank the reviewer for the careful comment. We experimentally examined the calibration dataset using the full calibration procedure with 100-fold averaging, acquired between 12:35:51 and 13:15:21. This corresponds to 23 s for one complete calibration of all 128 channels, or approximately 180 ms per channel when sweeping 32 voltage points.

For each channel, the phase-shifter calibration is performed by sweeping 32 voltage points to fit a cosine curve and thereby characterize the phase–voltage relationship. On a per-channel, per-voltage basis, approximately 0.08 ms is required for the intrinsic phase-shifter response to change its state, while the remaining ~5.55 ms is primarily consumed by photodiode (PD) readout, data communication between the RFSoC and the computer, and updating the phase-shifter control voltage. As a result, the total calibration time per channel is

$$(0.08+5.55)\ \text{ms} \times 32 = 180\ \text{ms}$$

In the experiment, each voltage point is measured 7 times and averaged to improve robustness. As a result, the effective calibration time is 1.4 s per channel, leading to a total calibration time of approximately 3 minutes for all 128 channels with 7-times averaging.

We elaborate on the timing contributions below:

1. **Intrinsic phase-shifter response limit.** The modulation speed of the thermo-optic phase shifter fabricated at imec is measured to be 12.5 kHz, corresponding to a response time of approximately 0.08 ms per state change. When sweeping 32 voltage points, the intrinsic lower bound for phase-shifter actuation is therefore about 2.5 ms per channel for all 32 voltages change.
2. **Calibration procedure for a single channel.** The calibration process proceeds as follows: (i) initialize the voltages for all 128 channels; (ii) select one channel and sweep 32 voltage points while reading out the PD signal; (iii) transfer the data to the computer for interpolation and cosine-curve fitting to identify the voltage corresponding to the peak optical; (iv) send the calibrated voltage back to the RFSoC to update the phase shifter; and (v) move to the next channel.
3. **Potential reduction toward the intrinsic limit.** The dominant overhead arises from data communication between the RFSoC and the computer. In principle, the fitting and peak-selection process could be implemented directly within the RFSoC, as the number of voltage points is limited and well within its processing capability. This would significantly reduce the overall calibration time and allow operation close to the

intrinsic phase-shifter limit. To remain conservative, we now describe the achievable calibration speed as 10 ms rather than quoting 2.5 ms as a strict bound.

4. **Relevance of calibration speed for system operation.** In a solid-state LiDAR system, calibration is not continuously required during normal operation. Beam steering relies on a pre-calibrated look-up table, and re-calibration is only performed during system initialization or if performance degradation is observed. Based on our experimental results, once calibrated, the OPA remains stable for at least half a year without requiring re-calibration.
5. **Relation to frame rate and FPA operation.** In FPA-based LiDAR systems, increasing angular resolution will influence the achievable frame rate. This motivates our parallel readout approach, which balances maximum ranging distance and frame rate. In addition, the current implementation employs thermo-optic phase shifters, which limit the achievable modulation speed; substantially higher frame rates are expected if electro-optic or MEMS-based modulators are adopted.
6. **Clarification of Extended Data Fig. 4.** The reported time of 1.4 s corresponds to a seven-times-averaged calibration for a single channel, as explained above. We have revised the manuscript to clarify this point.

Revision: To address this question, we have revised the text in the manuscript. Please check lines 127 to 138 and Extended Data Fig.5 caption.

Revised manuscript: line 127 to 138:

"As shown in Extended Data Fig. 1a-d, each OPA channel has a low-power thermo-optic phase shifter, providing beam steering with an efficiency of 6.4 mW/π phase shift and a modulation bandwidth of 12.5 kHz [13, 27]. Integrated phase interrogators placed between adjacent channels enable rapid on-chip phase calibration. In the current implementation, the calibration requires approximately 180 ms per channel to sweep 32 voltage points and extract the phase-voltage relationship, with the present limitation mainly imposed by the data readout capability [28, 29]. In principle, the calibration time can be further reduced to roughly 10 ms. It is important to note that the calibration algorithm does not run continuously during normal data acquisition. Beam steering relies on a pre-calibrated look-up table, and re-calibration is only performed during system initialization or if performance degradation is observed. Therefore, the above calibration times do not affect the normal operation of the LiDAR system."

Revised manuscript: Extended Data Fig.5 caption:

"Extended Data Fig. 5: Microscope images of the biaxial LiDAR components. a, SiN MMI tree fabricated using PECVD SiN, distributing optical power from a SiN bus waveguide into the 128-channel waveguides. The SiN bus waveguide sustains an optical power of 60 mW in the experiment, which is evenly split into 128 SiN waveguides and subsequently converted to Si waveguides for energy-efficient phase tuning through SiN-Si transitions, enabling high-power operation of the OPA. b, Energy-efficient optical phase-shifter array. c, Phase-interrogator array used for rapid on-chip calibration, requiring 1.4 s per channel with 7 repeated measurements and averaging to enhance robustness. d, Short grating emitters forming the line-shaped far-field of the OPA transmitter. e, Reconfigurable MZI-based photonic switch array, where each MZI integrates a PD monitor for ON/OFF state verification. f, 2D coherent pixel array of the FPA receiver."

Q5: Similarly, it is desirable to provide a detailed time breakdown of the reported frame rate, comparing potential limiting factors, including OPA direction-to-direction scanning time, transceiving time, laser chirp rate, electronics configuration, read-out time, and processing

time. Only by specifying each limiting factor can the authors support their claim that a 62 fps frame rate is potentially achievable.

Reply: We thank the reviewer for this valuable suggestion. The previously reported frame rate of 62 fps represents an idealized estimate that considers only the chirp duration and does not account for additional system-level timing overhead. In a more realistic assessment based on a detailed timing-budget analysis, we have revised the maximum achievable frame rate from 62 fps to 41 fps. The detailed time consumption for each frame is summarized below.

1. Thermo-optic switching time. The OPA beam steering is controlled using a pre-calibrated look-up table. The ON-OFF switching time of the thermo-optic phase shifter is approximately 80 μs per row, corresponding to a total switching time of 2.6 ms for 32 rows.
2. Chirp acquisition time. Once a specific row is activated, a complete chirp is transmitted and received before switching to the next row. Each row requires a full chirp duration of 500 μs. Therefore, the total chirp acquisition time for 32 rows is 16 ms.
3. Data processing and visualization in the current experimental setup. In our laboratory demonstration, the RFSoC performs data acquisition and FFT processing, while the processed data are transferred to a computer for point-cloud reconstruction and 3D visualization using Python. This computer-side data transfer, point-cloud generation, and 3D plotting introduce an additional overhead of approximately 76 ms per frame. We note that this overhead is mainly associated with the experimental visualization pipeline and is not intrinsic to the LiDAR hardware itself.

Here is a summary table with the RFSoC (Xilinx Zynq Ultrascale + RFSoC ZU29/39/49DR SOM) that we use in the experiment.

Stage	Median Time
32-row thermos-optic switch	2.6 ms
32-row chirp acquisition	16 ms
ADC to programmable logic streaming	1 ms
RFSoC 2048-point FFT for 32×16 pixels	2 ms
Peak acquisition and data storage	3 ms
RFSoC data packaging	6 ms
RFSoC-to-computer transfer	24 ms
computer receive + unpack	16 ms
computer point-cloud generation	10 ms
Python 3D plotting refresh	20 ms

In a practical embedded LiDAR system, such as an automotive LiDAR used for autonomous driving, the full 3D visualization on a computer is not required. Instead, the FFT output can be further processed directly on the embedded processor, FPGA, or ASIC to perform peak detection, range/intensity extraction, and point-cloud packet generation. These operations

are lightweight compared to computer -based 3D plotting and can be pipelined with the chirp acquisition. Therefore, only the acquisition-related time marked in green, together with a small embedded processing overhead, is required in a real-time implementation. Based on this timing budget, the minimum frame period is dominated by the 32-row chirp acquisition (which is 16 ms, corresponding to 62 fps). Considering a small additional overhead for embedded post-processing and frame packetization, a frame period of approximately 24.6 ms is feasible, corresponding to a potential frame rate of 41 fps, Therefore, although the current laboratory demonstration includes additional computer -based data transfer and visualization overhead, the underlying LiDAR hardware and embedded processing pipeline support a projected frame rate of approximately 41 fps in an optimized real-time implementation.

Revision: In order to clarify this question on the frame rate, we have revised the manuscript text. Please check line 36, lines 102 to 107, and lines 238 to 241.

Revised manuscript: line 36:

"Parallel point-cloud at 10 frames per second (fps) is demonstrated, while acquisition rates of up to 41 fps are supported by the architecture."

Revised manuscript: lines 102 to 107:

"Benefiting from the line-by-line scanning scheme, the prototype experimentally achieves parallel point-cloud acquisition at 10 frames per second (fps), currently limited by data-transfer and computer based processing throughput. Based on the timing budget, an optimized embedded implementation that eliminates the computer-side point-cloud reconstruction and 3D visualization overhead can support frame rates of up to 41 fps."

Revised manuscript: lines 238 to 241:

"The LiDAR prototype experimentally demonstrates a frame rate of 10 fps, which is currently limited by the data-transfer and computer-based processing throughput. However, the architecture theoretically supports acquisition rates of up to 41 fps, even when using relatively slow thermo-optic phase shifters"

Q6: In line 146, the authors wrote that the BPD includes two silicon diodes for cross-point addressing. Can they elaborate on the functionality of these two diodes and the diode attached to the phase interrogation PD in their OPA? It appears that diodes are used to enable and disable PDs with DC biasing, but the current manuscript is not explicit enough.

Reply: We thank the reviewer for the insightful comment. The integrated Si diodes serve two essential functions for both the photodiodes (PDs) in the balanced photodetectors (BPDs) and the phase interrogation photodiodes.

1. the Si diodes enable cross-point addressing, which significantly reduces the required number of bond pads and improves scalability within a given chip area.
2. the Si diodes suppress crosstalk from the cross-point addressing.

A more detailed explanation is provided below.

For the **phase interrogators PD in OPA**:

1. With Si-diode-assisted cross-point addressing, the 128 phase interrogation channels are organized into eight blocks, each comprising 16 channels. This architecture reduces the number of bus metal interconnects and corresponding bond pads from 256 (128 × 2), required for independent control of all phase interrogators, to only 24

(16 column lines plus 8 row lines). This substantially enhances the scalability of the system.

2. A known drawback of cross-point addressing is the potential for crosstalk caused by sneak-current paths and dark-current leakage through unselected channels. For instance, without proper electrical isolation, currents from neighbouring channels such as CH2 and CH17 may leak into CH1. The integrated Si diode in each channel blocks these unintended current paths, thereby ensuring effective electrical isolation and maintaining the stability and accuracy of the phase calibration process.

For the **BPDs in FPA**:

1. Reduction of bond pads. Taking the current 32 × 16 BPD array as an example, cross-point addressing drastically reduces the number of required bond pads. For independent control and readout of each BPD, a total of 32 × 16 × 3 bond pads would be required. In contrast, with the cross-point parallel readout scheme, the number of bond pads is reduced to 32 × 2 + 16, corresponding to row and column lines for parallel operation. This represents an approximate reduction of bond-pad scaling from the N^2 level to the N level, which significantly improves system scalability.
2. Suppression of inter-channel and inter-pixel crosstalk. The integrated Si diodes play a critical role in suppressing both inter-channel and inter-pixel crosstalk. When a specific row is activated, 16 BPD channels are read out simultaneously. Without proper electrical isolation, sneak-current paths can form among channels, leading to inter-channel crosstalk and distortion of the differential current measured by each BPD. In addition, the ON-OFF crosstalk of the BPDs is measured to be −30 dB, as shown in Extended Data Fig. 7(f). This indicates that, regardless of whether all pixels are activated or not, inter-pixel crosstalk and dark-current leakage from unselected pixels can still influence the measured differential current. The Si diodes enforce a well-defined current direction within each pixel and effectively block leakage currents from other pixels, particularly those flowing in the reverse direction, thereby ensuring accurate differential readout.

<u>Revision</u>: In order to clarify this point, we have revised the text in the manuscript. Please check lines 139 to 144 and Extended Data Fig.1e.

Revised manuscript: lines 139 to 144:

"To reduce the routing complexity, the 128 OPA channels are divided into 8 blocks of 16 channels and addressed using a cross-point scheme, reducing the number of bus metal wires from 256 (128 × 2) for independent phase-interrogator control to 24 (16 + 8). In addition, integrated Si diodes provide electrical isolation by suppressing sneak-current paths and dark-current leakage from unselected photodiodes, thereby mitigating inter-channel crosstalk, as illustrated in Extended Data Fig. 1e."

Revised manuscript: Extended Data Fig.1e:

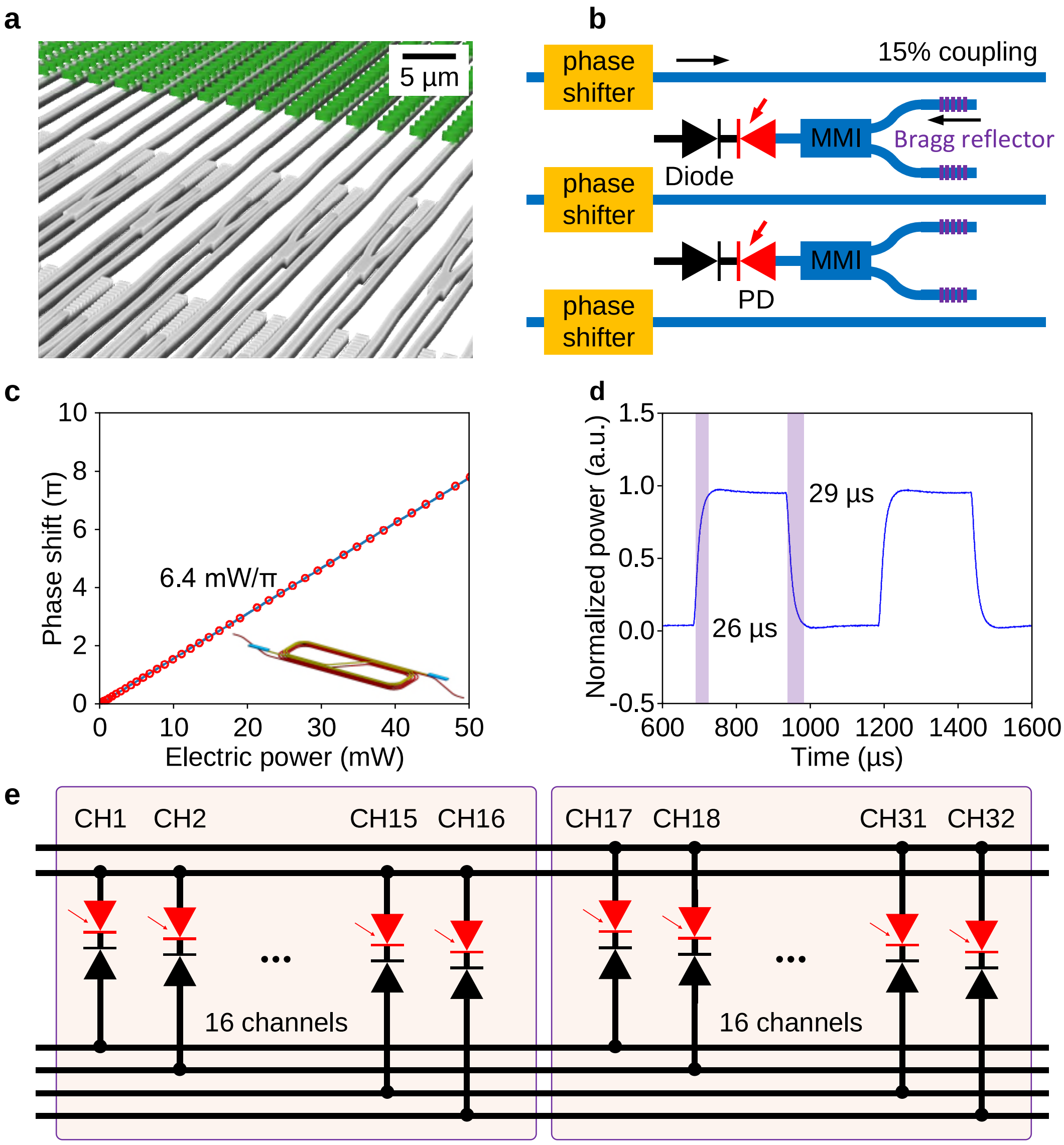

"Extended Data Fig. 1: Design and characterization of the OPA transmitter. a, Schematic illustration of the short-grating emitter and the on-chip rapid phase interrogators. b, Design of the phase interrogator. c, Measured power consumption of the thermo-optic phase shifter in the OPA transmitter, showing 6.4 mW/π tuning efficiency. d, Measured rise and fall times of the phase shifter, corresponding to an OPA beam-steering modulation bandwidth of 12.5 kHz. e, The 128 OPA channels are divided into 8 blocks of 16 channels. Cross-point addressing reduces the number of bus metal wires from 256 (128 × 2), required for independent phase-interrogator control, to 24 (16 + 8). Integrated Si diodes provide electrical isolation by suppressing sneak-current paths and dark-current leakage from unselected photodiodes, including neighbouring channels such as CH2 and CH17 that would otherwise couple into CH1, thereby mitigating inter-channel crosstalk."

Q7: In lines 148 and 149, the authors wrote that the receiving angles vary across channels but are identical across rows within each channel. Can they elaborate on why the receiving angles are identical across rows within each channel? Is this a physical fact or a design limitation? Ideally, the diffraction from each grating should point toward the lens center, resulting in different receiving angles across the array.

Reply: We are grateful to the reviewers for their careful review. Indeed, in the ideal case, the receiving angle of each grating is designed to align with the chief ray of the lens. This can be achieved by engineering different receiving angles across channels and by physically rotating the grating orientation across different rows. Such an approach becomes essential for extremely large-scale pixel arrays, where precise matching between the grating receiving and the lens chief rays is critical.

There are two main considerations that motivate our decision not to rotate the gratings to align with the chief ray of the lens.:

1. In the present work, this additional level of grating rotation is not mandatory, as the lens aperture is much larger than the overall pixel array size. Our primary objective here is to verify whether gratings with both positive and negative receiving angles can operate reliably within the proposed architecture. Therefore, we designed different receiving inclination angles across channels without implementing row-dependent grating rotation.
2. Each row of pixels in the focal-plane array receives a line-shaped beam pattern from the OPA with a defined polarization state. Rotating the gratings could therefore introduce a polarization mismatch with the OPA transmitter.

Revision: In order to clarify this point, we have revised the text in the manuscript. Please check lines 169 to 175.

Revised manuscript: lines 169 to 175:

"As shown in Extended Data Fig.3a-c, the receiving inclination angles are varied across channels to match the chief-ray angular range of the lens, but are kept identical across rows within each channel to preserve the polarization alignment with the line-shaped OPA emission. This design reduces polarization mismatch with the transmitter while enabling each channel to collect light over the appropriate angular range, thereby increasing the effective aperture."

Q8: Following up on comment 7, can the authors elaborate on how they map the receiving angular range of 5° to -5° to their 16° × 8° FOV? Is this determined by their lens design? Additionally, did they implement any measures to reduce inter-pixel crosstalk within the same row? For example, is there observable crosstalk between the central grating for near-normal receiving and its neighbors?

Reply: We thank the reviewer for the insightful comments. The field of view (FoV) in our system is determined by the combination of the lens focal length and the physical dimensions of the pixel array. Specifically, the 16° extent corresponds to the total length of the 32 rows, for which no physical rotation of the gratings is implemented, while the 8° extent corresponds to the total length of the 16 channels, across which the grating receiving angles are intentionally varied by tunning the teeth period.

The reviewer is correct that, in the initial theoretical design, the grating receivers were designed to cover an angular range from −4° to +4°. However, following fabrication, we measured the actual device dimensions and re-simulated the grating performance using the as-fabricated parameters. This post-fabrication analysis indicates that the effective receiving angular range of the gratings extends from −5° to +5°, which therefore represents the physically realized grating response reported in this work. The 1° deviation from the nominal design does not lead to a noticeable degradation in optical performance.

In the experiment, we did not observe any noticeable crosstalk. In our design, the FPA pixels operate exclusively in the receiving mode, and the received light collected by each grating is directly coupled into its corresponding BPD without propagating through neighbouring pixels. As a result, optical (photonic) crosstalk between adjacent gratings within the same row is not expected and was not observed experimentally. Potential electrical crosstalk arising from parallel readout within the same row (inter-channel crosstalk) is effectively suppressed by the integrated Si diodes inside each BPD, as discussed in detail in response to Q6. These diodes block unintended current paths and prevent leakage currents from neighbouring channels, thereby ensuring stable and isolated readout even when multiple BPDs are activated simultaneously.

Revision: In order to clarify this point, we have revised the text in the manuscript. Please check lines 175 to 177 and caption of Extended Data Fig. 3.

Revised manuscript: lines 175 to 177:

"The grating pitches were designed for a receiving angular range of −4° to 4°, but fabrication-induced deviations shift the actual range to approximately −5° to 5° with only minor additional optical loss."

Revised manuscript: Extended Data Fig. 3 caption:

"Extended Data Fig. 3: Design and characterization of the FPA receiver. a, Schematic illustration of the FPA receiver. By design, the grating receiver angles span from −4° to 4° to match the 8° FoV and ensure alignment of all gratings with the lens optical axis. Simulations based on the as-fabricated dimensions indicate an actual angular range of approximately −5° to 5°; however, this deviation introduces only a minor additional optical loss. b, Design of the grating pitch and corresponding simulated receiving angle for different channels. c, Simulated grating coupling efficiency and back-reflection for different receiver channels. Although the central channels with grating emission angles of ±1.5° exhibit high back-reflection, this does not affect their operation as receivers for free-space coupling into the photonic chip."

Q9: Can the authors comment on the total power consumption of their prototype, ideally breaking down into each sub-assembly, including the laser, OPA, FPA, RFSoC, and TECs?

Reply: Thank you for this valuable suggestion. We provide an approximate breakdown of the power consumption of the main sub-assemblies below.

1. **Laser**. The DFB laser (Thorlabs DFB1550P) operates at 1.15 V and 150 mA, corresponding to an electrical power consumption of approximately 0.17 W. The associated laser driver (Koheron CTL200 CB-600) introduces additional power consumption, including that required for its internal temperature stabilization (TEC) for laser. However, given the integrated nature of the driver module, a precise separation of these contributions is not readily accessible.
2. **OPA.** The power consumption of the optical phased array (OPA) arises from 128 phase shifters and 128 phase interrogators, and can be estimated as

$$128 \times (6\ \text{mW} + 1.65\ \text{V} \times 0.033\ \text{mA}) \approx 0.78\ \text{W}.$$

Here, 6 mW denotes the average consumption per phase shifter, while 1.65 V and 0.033 mA correspond to the operating voltage and photocurrent of each phase interrogator.

3. **FPA.** For the focal plane array (FPA), 16 balanced photodetectors operate simultaneously, yielding an estimated total power consumption of

$$16 \times (2 \times 1.65\ \text{V} \times 0.033\ \text{mA}) \approx 1.7\ \text{mW}.$$

4. **RFSoC and TEC.** The entire system is powered by a standard 12 V AC adapter. However, because the RFSoC and TEC are implemented as part of fully integrated and packaged subsystems, their individual power consumption is not directly accessible in the present experimental setup. Moreover, the TEC operates in a closed-loop mode to maintain a stable temperature of 20 ℃, such that its power consumption varies dynamically with the ambient conditions. We therefore regret that a reliable quantitative breakdown of the power consumption of the individual components cannot be provided for the current prototype.

Q10: The authors claim that their sigma-delta modulation (SDM) achieved precise phase control without giving the equivalent phase/bit resolution. Please support your claim quantitatively.

Reply: To quantify the resolution achieved by the sigma-delta modulation (SDM), we have computed the root-mean-square (RMS) phase noise after the thermal phase shifter, based on the in-band shaped quantization noise of a first-order SDM combined with a first-order thermal RC model of the phase shifter.

Using the parameters of the implemented system, namely P_π = 6 mW, B = 12.5 kHz (thermal bandwidth), F_{SDM} = 8 MHz (SDM update rate), and P_{max} = 50.7 mW (power delivered in the 'high' state, zero otherwise), yields an RMS phase noise of approximately 3.0° per phase shifter. This corresponds to an equivalent resolution of 8.7 bits ENOB full-scale. For comparison, under an idealized brick-wall low-pass assumption, the same SDM would yield an RMS phase noise of approximately 0.098°, corresponding to 13.6 bits ENOB full-scale. This clearly overestimates the achievable resolution, as it neglects the gradual first-order roll-off of the thermal response of the phase shifter. At the array level, the optical phased array (OPA) consists of 128 parallel phase shifters. As the operating point of each phase shifter differs due to process variation and beam steering, the SDM-induced phase noise at each element can be reasonably assumed to be uncorrelated. Under this assumption, the array-level RMS phase noise reduces to approx. 0.26° (3.0°/sqrt(128)), corresponding to sub-degree phase accuracy at the array level. Further optimization is possible by reducing P_{max}. For example, using $P_{max} \approx 3P_\pi$ still allows for a full 2π phase tuning range with margin while reducing the RMS phase noise by approximately a factor of three.
Revision: To address this point, we have revised the text in the manuscript. Please check lines 400 to 401.

Revised manuscript: lines 400 to 401:

"High-resolution phase control in the OPA transmitter and the MZI-tree was implemented using sigma-delta modulation with a calculated root-mean-square (RMS) phase noise of approximately 3° per phase shifter."

Q11: In lines 172 and 173, the authors wrote that the signals are then anti-aliasing filtered. Can they elaborate on this procedure and its purpose?

Reply: We thank the reviewer for raising this point and for seeking clarification on the anti-aliasing filtering procedure. In our system, the anti-aliasing filtering is implemented in the analog domain between the TIAs and the ADCs.

The anti-aliasing stage was implemented as a 3rd-order inverse Chebyshev low-pass filter with a cutoff frequency of 30 MHz. The primary purpose of this filtering is to suppress high-frequency components prior to digitization. Without sufficient attenuation, these components could alias during ADC sampling, meaning that spectral content located outside the useful signal band would be folded (shifted) into the lower-frequency beat-frequency region used for distance and velocity extraction.

The dominant out-of-band components originate from:

1. The broadband noise of the photodetectors and TIAs.
2. The high-frequency quantization noise associated with the sigma-delta modulation used to drive the OPA phase shifters.

Although these components do not contain useful ranging or velocity information, after sampling they could overlap with the desired baseband signals and degrade the accuracy of the subsequent FFT-based processing.

Therefore, the anti-aliasing filter limits the analog signal bandwidth prior to ADC conversion, preventing spectral folding of out-of-band noise into the measurement band, improving the effective signal-to-noise ratio, and ensuring stable and accurate extraction of distance and velocity information during parallel acquisition enabled by spatial multiplexing.

Revision: We updated the text in the manuscript. Please check line 198**.**

Revised manuscript: line 199:

"The resulting signals are then anti-aliasing filtered by a third-order inverse Chebyshev low-pass filter with a cutoff frequency of 30 MHz, digitized by analog-to-digital converters (ADCs) in the RFSoC, and subsequently processed using fast Fourier transform (FFT)-based DSP to extract distance and velocity information."

Q12: Can the authors comment on how many points are stored in their 4 kB ADC register for a 500 μs chirp, and how they determine the chirp flip point (i.e., from up-chirp to down-chirp or vice versa) in their stored data?

Reply: For a 500 μs triangular chirp waveform, 2048 sampling points for each pixel readout are stored in the 4 kB ADC register.

With respect to the determination of the chirp flip point, the FMCW waveform generation and the ADC sampling are both synchronized and controlled by the RFSoC. As a result, the temporal structure of each chirp, including the up-chirp and down-chirp segments and their transition point, is predefined by the system timing rather than inferred from the sampled data. The flip point therefore corresponds to a fixed and known sampling index within the stored 2,048 points.

During digital signal processing, the RFSoC uses this predefined timing information to accurately separate the up-chirp and down-chirp portions of the acquired data. No additional post-processing or slope-based detection is required to identify the chirp direction change, and the ADC always captures the complete chirp waveform for each acquisition.

Revision: We updated the text in the manuscript. Please check line 225 and Fig. 3(b) caption**.**

Revised manuscript: line 225:

"For each pixel, the temporal waveform is recorded in a 4 kB ADC register using a 500 μs triangular chirp waveform, sampled with 2048 points (Fig. 3b)."

Revised manuscript: Fig. 3(b) caption:

"Fig. 3: Characterization of 3D imaging results. a, Full-frame readout data. 16 channels are read out simultaneously during line-by-line scanning across 32 rows, with the readout data of each channel stored in a 4 kB register. b, Pre-distorted triangular chirp waveform with a duration of 500 μs and a maximum bandwidth of 3 GHz, and a representative pixel read-out signal. Each 500 μs triangular chirp waveform is sampled with 2048 points. c, Representative FFT spectrum from a receiver pixel, indicating a target distance of 1 m and an SNR of 27 dB. The up-chirp and down-chirp peaks overlap due to the static target. d, Reconstructed 3D imaging of the "imec" logo at 1 m."

Q13: It appears that they are measuring targets coated with reflective tape. They reported a reflectivity of ~70% at 1550 nm. Can they elaborate on how this reflectivity is measured?

Reply: We thank the reviewer for raising this question. The reflective tape used in our experiments is a commercially available retroreflective tape for traffic signs (A large-scale microelectromechanical-systems-based silicon photonics LiDAR" (Nature, 2022)). The reported reflectivity of approximately 70% is based on the manufacturer's specification, which is provided for the visible wavelength range and not for 1550 nm. We did not independently measure the absolute reflectivity of the tape at 1550 nm, nor did we intend to imply that the quoted value corresponds to the operating wavelength of our LiDAR system.

In our experiments, the reflective tape is primarily used as a high-contrast target to ensure sufficient return signal for system evaluation and demonstration, rather than for absolute reflectivity calibration. As such, the reported reflectivity value serves only as a qualitative reference to indicate that the target is highly reflective, and the LiDAR performance metrics reported in this work (such as range, velocity extraction, and imaging capability) do not rely on an absolute knowledge of the target's reflectivity at 1550 nm.

To avoid potential confusion, we will clarify in the revised manuscript that the 70% reflectivity value is based on the manufacturer's specification of the commercial tape, rather than a calibrated measurement at the LiDAR operating wavelength.

Revision: We updated the text in the manuscript. Please check lines 229 to 230.

Revised manuscript: lines 229 to 230:

"The reflective target is made of a material similar to that used in traffic signs [18]."

Q14: In Extended Data Fig. 5, the linearization is performed with a chirp rate of 1 kHz (1 ms), whereas the chirp signal shown in Fig. 3(b) has a duration of 500 µs. Please clarify whether two chirp rates are used or whether the ADC captures only part of the chirp.

Reply: We thank the reviewer for pointing out this potential ambiguity and clarify that two different chirp durations are indeed used for different experimental purposes. A 500 µs triangular chirp waveform is employed for the 3D imaging experiment at 1 meter, which is shown in Fig. 3(b). In addition, a 1000 µs triangular chirp waveform is used for the 20-m range measurement in order to further verify the effectiveness of the proposed chirp linearization method for longer acquisition time. Extended Data Fig. 5 presents the chirp linearization results for the 1000 µs chirp used in the 20-m range experiment. No partial chirp acquisition is involved; in each case, the ADC captures the full chirp corresponding to the specific experimental configuration.

Revision: To clarify this point, we have revised the text in the manuscript. Please check lines 244 to 245 and Extended Data Fig. 6 caption.

Revised manuscript: lines 244 to 245:

"Measurements are performed with a 35 mm focal-length lens (f/1.4) and a triangular chirp waveform of 1000 μs which is sampled with 4096 points, yielding a FoV of 3.6° × 1.8°."

Revised manuscript: Extended Data Fig. 6 caption:

"Extended Data Fig. 6: Chirp linearization schematic and characterization. a, Schematic of chirp linearization for a directly modulated FMCW laser. A 35 m fiber loop is used to emulate the round-trip propagation delay of a target located at 25 m in air. An AWG generates the chirp ramp and provides the trigger signal for oscilloscope synchronization. The Hilbert transform is applied to extract the instantaneous phase of the beat signal, which is compared with the ideal chirp ramp generated by the AWG to evaluate the frequency error. The index i denotes the iteration number, and N_{loop} represents the total number of iterations. VOA, variable optical attenuator. b, Measured frequency error over the chirp duration versus iteration number. Each 1000 μs triangular chirp waveform is sampled with 4096 points. After four iterations, the maximum frequency deviation is reduced from 250 MHz to 1.5 MHz over a 3 GHz sweep, corresponding to a normalized frequency error of 0.05%.

Reviewer #2: This manuscript presents a highly innovative, monolithically integrated biaxial FMCW LiDAR system, cleverly combining an OPA transmitter and a coherent FPA receiver—comprising over 4,500 components—on a single silicon photonic chip. This biaxial architecture successfully circumvents the reliance on on-chip optical circulators and achieves 3D imaging at a single wavelength without broadband tuning, marking an impressive step forward in the chip-level integration of solid-state LiDAR. The prototype packaging and system-level demonstrations also exhibit a high standard of engineering. However, to meet the rigorous publication standards of this journal and to genuinely substantiate the system's practical value in "dynamic scene perception," the manuscript contains several critical flaws regarding its velocity calibration methodology, presentation of raw data, and validation of high-frame-rate imaging quality. Therefore, I recommend a Major Revision to address the following issues before a final decision can be made.

Reply: We are grateful for the comments from the reviewer and for the chance to further improve the impact of our work.

Q1: the methodology for calibrating the velocity resolution lacks metrological rigor. The authors utilized a "square-board target with spatially varying motion" in Extended Data Fig. 6 to claim a 1 mm/s velocity resolution, yet they failed to establish any ground truth for the physical velocities across different regions of this target. A qualitative color heat map alone cannot rule out whether these minute velocity variations stem from low-frequency phase noise, ambient mechanical vibrations, or algorithmic artifacts. I strongly advise the authors to perform quantitative testing using a known, precisely calibrated moving target (e.g., a high-precision motorized linear translation stage or a spinning disk with a known angular velocity) and to provide a statistical analysis encompassing multiple velocity steps to scientifically and irrefutably validate their velocity resolution limit.

Reply: We sincerely thank the reviewer for the constructive suggestion regarding the metrological rigor of the velocity resolution calibration.

In response, we have spent significant time to perform additional quantitative experiments using a well-defined and precisely controlled moving target. Specifically, we employed a rotating turntable with a known and constant angular velocity of 1 rpm (0.105 rad/sec), on which a rectangular board was mounted as the scattering object. This setup provides a reliable and traceable ground truth for velocity across different spatial locations on the object.

Based on the known angular velocity, the linear velocity distribution across the surface can be accurately calculated as a function of the radial distance from the rotation center. We then compared these theoretical velocities with the measured values obtained from our system. The results show strong agreement between the measured and expected velocities, thereby providing quantitative validation of the system's velocity measurement capability.

Furthermore, we analysed the velocity sensitivity and resolution by examining the minimum detectable velocity variations across multiple spatial regions and repeated measurements. The experimental results confirm that the system is capable of resolving velocity changes on the order of ~1.36 mm/s, while excluding contributions from low-frequency phase noise or environmental perturbations.

<u>Revision</u>: In order to better address this question, we revised the text and added a figure. Please check lines 256 to 259 and Extended Data Fig. 7.

Revised manuscript: lines 256 to 259:

"The velocity resolution was further evaluated using a rectangular board mounted on a rotating stage to generate spatially varying motion. As shown in Extended Data Fig. 7, velocity differences of approximately 1.36 ±0.08 mm/s were clearly resolved."

Revised manuscript: Extended Data Fig. 7:

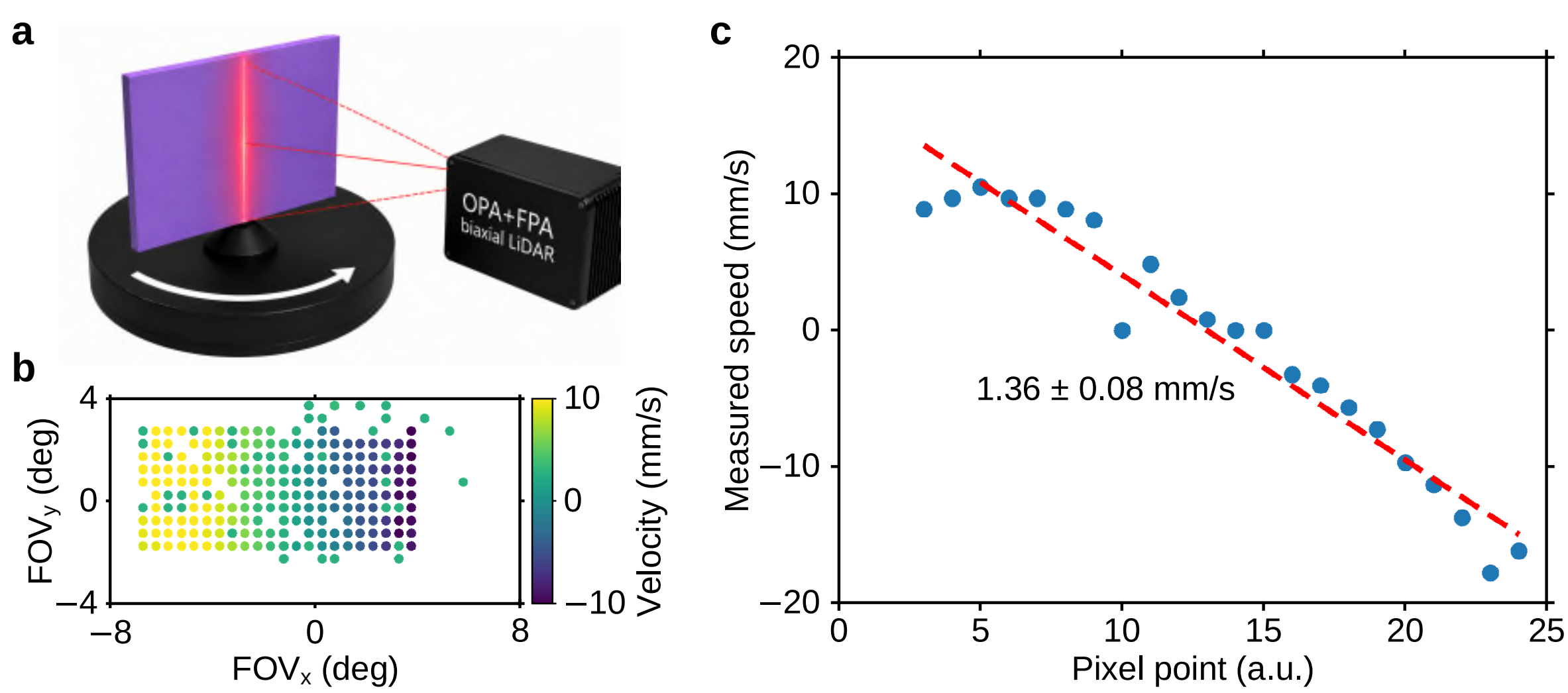

"Extended Data Fig. 7: Velocity resolution measurement. a, Experimental setup of a rectangular board of 20 cm width mounted on a rotating stage at 1 revolutions per minute (rpm). The LiDAR prototype was positioned 1.1 m away. b, The Velocity point cloud measured from the rectangular board. c, The measured velocity resolution is 1.36 ± 0.08 mm/s, in reasonable agreement with the theoretical value of approximately 0.8 mm/s."

Q2: The presentation of the point cloud data involves artificial modifications that obscure the system's true signal-to-noise ratio (SNR) and detection performance. When demonstrating

the 20-meter 3D imaging in Fig. 4b, the authors artificially added a purple virtual background point cloud behind the target, conceding in the text that this was included solely to "enhance the visual contrast." In a rigorous hardware characterization paper, implanting non-physical, virtual data is unacceptable, as it severely misleads the reader regarding the system's true field of view, point cloud sparsity, and background noise distribution. The authors must remove all virtual point clouds in the revised manuscript and present the raw, unaltered measurement results to objectively reflect the true imaging quality without the aid of a high-contrast artificial background.

<u>Reply</u>: We thank the reviewer for this important comment. We agree that artificial point clouds may obscure the true system performance. In the revised manuscript, all virtual/background point cloud elements have been removed. The updated figures now present only the raw, unaltered measurement results, providing a more accurate representation of the system's imaging quality and noise characteristics.

<u>Revision</u>: Please check lines 247 to 248 and Fig. 4b**.**

Revised manuscript: lines 247 to 248:

"displaying only detected points, with the FFT peak 25 dB above the baseline"

Revised manuscript: Fig. 4b:

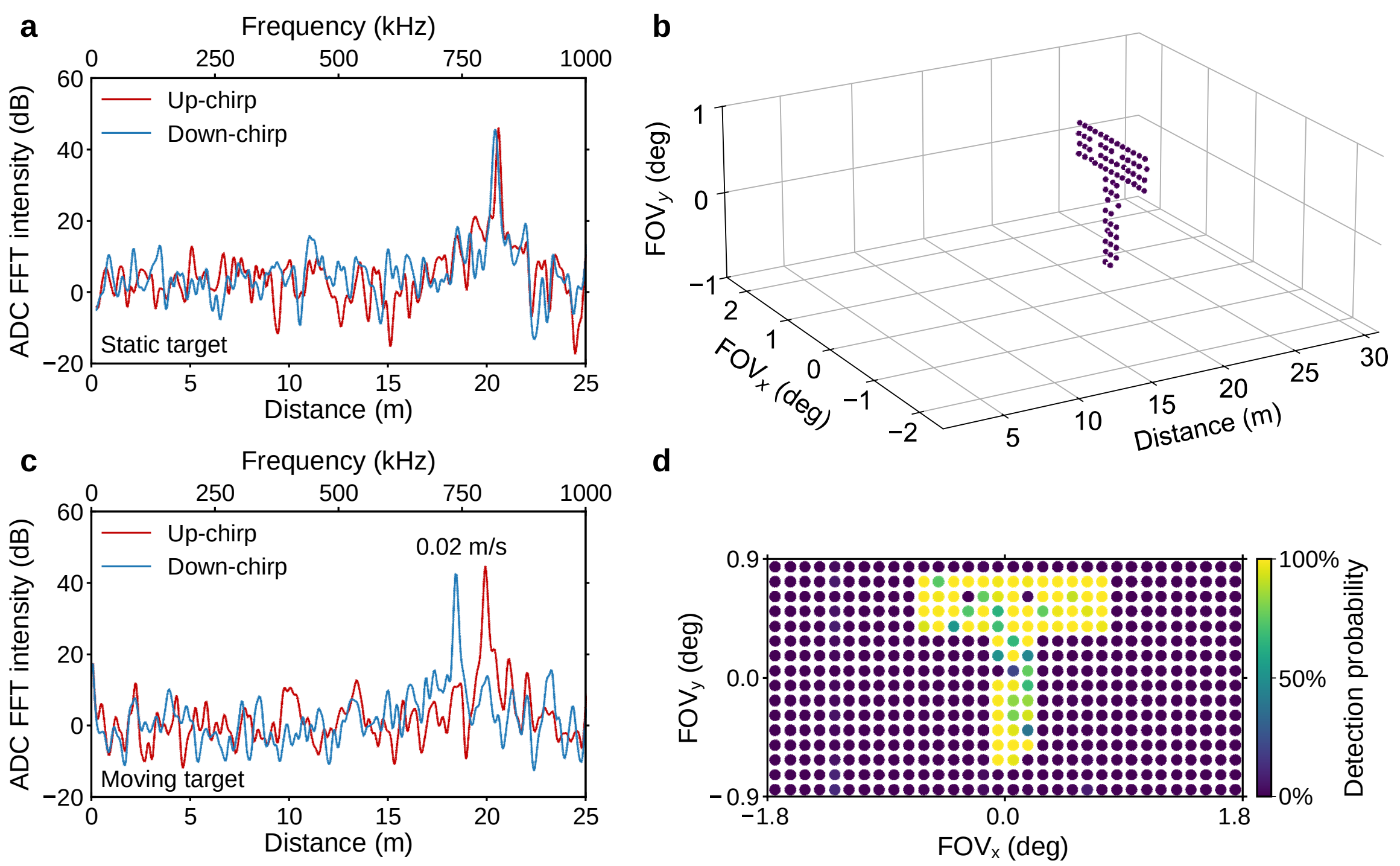

"Fig. 4: Characterization of 20 m ranging and Doppler velocimetry. a, FFT spectrum acquired from a representative receiver pixel, revealing the beat-frequency peak corresponding to a static "T" target positioned at a distance of 20 m. b, Reconstructed point cloud of the "T" target from a single, non-averaged frame. c, FFT spectrum from a representative receiver pixel for a moving target with a velocity of 0.02 m/s. The target is approaching the LiDAR prototype from 20 m to 19 m, as indicated by a higher beat frequency in the up-chirp compared with the down-chirp. d, Detection probability evaluated over 100 accumulated frames, demonstrating robust target detection."

Q3: There is a distinct disconnect in the validation linking high frame-rate operation to actual single-frame imaging quality. Although the paper claims an experimental scanning rate of 10 fps, the evaluation of the detection probability for the target at 20 meters (Fig. 4d) relies on "100 accumulated frames." This inadvertently reveals that under the extremely short single-frame dwell time at 10 fps—restricted by the weak emitted power (3.7 mW)—the true SNR of a single scan is highly likely insufficient for effective imaging. To truly prove that the system is capable of dynamic scene perception, the authors cannot rely on static accumulation to mask poor single-frame quality. They must provide raw, single-frame dynamic point clouds captured at the nominal 10 fps continuous scanning rate, demonstrating that the system can output clear, complete, and usable point clouds without severe motion blur within a short integration time.

<u>Reply</u>: We thank the reviewer for this insightful comment. We would like to clarify that Fig. 4b shows single-frame point cloud results, obtained at the nominal scanning rate (10 fps), rather than from any multi-frame accumulation.

The "100 accumulated frames" mentioned in Fig. 4d are used solely for statistical evaluation of detection probability, and are not used to generate or enhance any 3D point cloud results. Therefore, the reported imaging performance in Fig. 4b reflects the true single-frame measurement capability of the system without temporal averaging.

We have clarified this distinction explicitly in the revised manuscript to avoid any misunderstanding. For completeness, we also show in Fig. R1 (not added to the manuscript) 4 sequential acquired frames (without any averaging). This indicates that each frame is able to provide usable point clouds that can be used for dynamic scene perception.

<u>Revision</u>: We revised the text in the manuscript. Please check lines 246 to 248 and Fig.4b.

Revised manuscript: lines 246 to 248:

"and the reconstructed three-dimensional image at 20 m from a single non-averaged frame is shown in Fig. 4b, displaying only detected points, with the FFT peak 25 dB above the baseline."

Revised manuscript: Fig. 4b: please check from last Q&A (Q2).

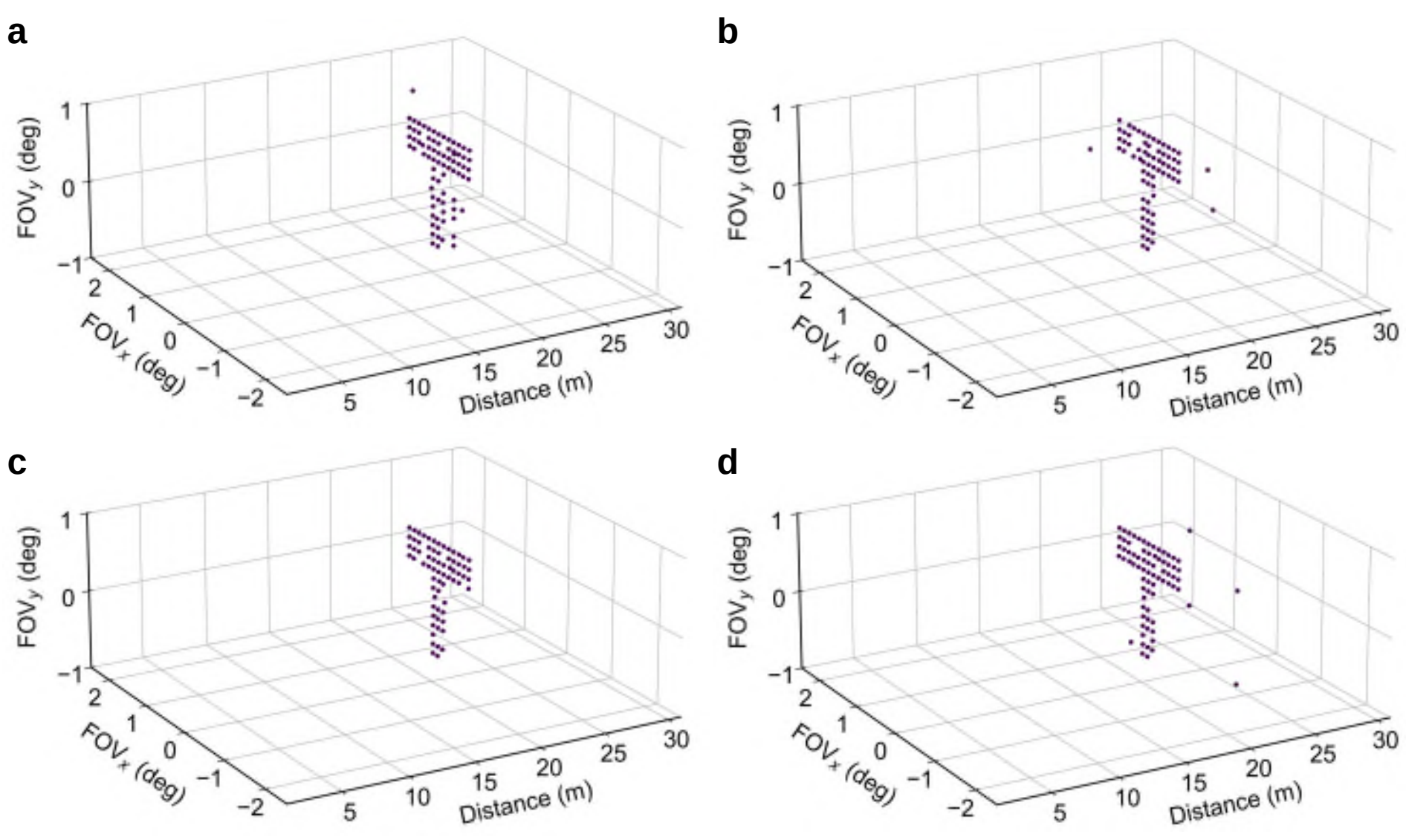

Fig. R1: Point cloud of four sequential frames, chronologically following the frame shown in Fig. 4d.

Q4: The current test scenarios and target parameters are overly idealized, failing to adequately support the claim of a "practical system-level approach." The current near-distance ranging experiment utilized a planar target with an exceptionally high reflectivity of 70%, which is a far cry from the stringent optical conditions of real-world industrial applications (e.g., autonomous driving systems encountering 10% reflectivity from dark vehicles or clothing). The authors should supplement the manuscript with an SNR evaluation and maximum range demonstration using standard low-reflectivity targets (e.g., 10%). Furthermore, it is recommended to introduce test targets with complex 3D morphologies, depth variations, and a certain degree of ambient light interference. Showcasing the actual measurement results under these conditions would far more convincingly validate the core advantages of this coherent FMCW architecture in terms of crosstalk immunity and 3D spatial resolving power.

Reply: We thank the reviewer for these valuable and thoughtful suggestions. Our response is based on the following two aspects.

1. The ~70% reflectivity target used in our experiments corresponds to materials commonly used in traffic signs, and similar targets are also widely adopted in related works for consistent benchmarking under controlled conditions. ("*A large-scale microelectromechanical-systems-based silicon photonics LiDAR*" (Nature, 2022), "*A universal 3D imaging sensor on a silicon photonics platform*" (Nature, 2021), and "*Frequency–angular resolving LiDAR using chip-scale acousto-optic beam steering*" (Nature 2023))

2. We agree that low-reflectivity scenarios are important for practical applications. In this work, however, our primary focus is on demonstrating the fully integrated monolithic OPA+FPA architecture, and the current system has not yet been fully optimized in terms of optical loss and emitted power. Moreover, for the same 20 m range, the reduced reflectivity (e.g., 10%) can in principle be compensated by increasing the optical power. Notably, our SiN platform still has substantial power handling margin, leaving significant room for performance improvement in future iterations.

We greatly appreciate the reviewer's insightful and professional comments. Although we are currently limited in reproducing all the suggested scenarios, we will prioritize these aspects in our future work and carry out more comprehensive evaluations accordingly.

Best regards,

Huaqing Qiu, on behalf of all authors

21st June 2026
